# Computational identification of rare pathogenic genomic variants in esophageal cancer markers: Transcript-level analysis, sequence-based insights, and structural-functional impacts of non-synonymous SNPs

**DOI:** 10.1016/j.bbrep.2026.102503

**Published:** 2026-02-23

**Authors:** Muhammad Bilal Azmi, Sajida Qureshi, Rafia Wasi, Saad Khalid Niaz, Syed Danish Haseen Ahmed

**Affiliations:** a Computational Biochemistry Research Laboratory, Department of Biochemistry, Dow Medical College, Dow University of Health Sciences, Karachi, 74200, Pakistan; b Department of Surgery, Dow Medical College, Dow University of Health Sciences, Karachi, Pakistan; c Sindh Institute of Advanced Endoscopy and Gastroenterology (SIAG), Heritage Building 1st Floor, Dr. Ruth K.M Pfau Civil Hospital, Baba e Urdu Road, Karachi, Sindh, 75000, Pakistan

**Keywords:** Esophageal cancer, nsSNPs, Oncogenic, Precision medicine, Transcript

## Abstract

**Background and aims:**

Esophageal cancer (EC) is a rapidly progressing malignancy that significantly contributes to cancer-related mortality. The genetic causes of EC, particularly rare coding pathogenic variants, remain incompletely defined. This study focuses on non-synonymous single nucleotide polymorphisms (nsSNPs) because they can impact the functions of critical proteins implicated in carcinogenesis.

**Methodology:**

We employed an extended computational framework for the identification and analysis of rare-coding nsSNPs within 28 EC-associated genes and identified 126 protein isoforms from public databases such as NCBI and ENSEMBL, focusing on those with MAF <1%. Functional predictions were evaluated using different bioinformatics tools to ascertain pathogenicity. Furthermore, ten rare-coding nsSNPs were mapped to 23 transcript-level variants within genes such as *GRB7, SLCO1A2, HIF1AN, KCNQ3,* and *DLL1*.

**Results:**

Computational analysis showed that these variants have a prominent impact on protein stability and function because substitutions occurred at conserved residues, thereby perturbing the stability and important regulatory attributes of the proteins. Structural modeling supported our findings: some variants may compromise domain integrity and influence the signal transduction pathways relevant to the progression of EC. The oncogenic potential of the conserved variants was also computationally validated using the Cscape tool, with candidates including *rs1591837395* (G68R) in *SLCO1A2* and *rs758624092* (D201H) in *HIF1AN*. Collectively, this study identified rare and potentially pathogenic coding variants in EC-related genes and elaborated on their effects at the transcript, sequence, and structural levels.

**Conclusion:**

Integrating these insights into EC provides an extended view of its molecular mechanisms and may be used in the near future as a basis for precision medicine for early diagnosis and targeted therapy.

## Highlights

• Rare deleterious nsSNPs in 28 esophageal cancer genes were screened computationally.
• *GRB7, SLCO1A2, HIF1AN, KCNQ3* and *DLL1* showed high-impact variants.
• Most variants affected conserved residues linked to structure and regulation.
• Stability, flexibility and RMSF analyses indicated domain or signaling disruption.
• Prioritized variants are proposed for experimental validation as biomarkers.

## 1 Introduction

EC is a highly aggressive malignancy of the gastroesophageal tract and is a significant contributor to global cancer mortality [1]. Recent epidemiological place EC among the top ten causes of cancer-related deaths worldwide, with a notably poor prognosis [2]. The five-year survival rate in most areas remains very low due to the late stages of diagnosis and the intrinsic aggressiveness of the disease [3]. The burden of EC varies geographically, and high-incidence “esophageal cancer belts” have been identified in parts of East Asia, Eastern and Southern Africa, and portions of Central Asia. This reflects the combined effects of environmental exposures, dietary patterns, and genetic predisposition [[4], [5], [6]].

Two histological subtypes predominate: esophageal squamous cell carcinoma (ESCC), arising from the epithelial lining of the esophagus and more prevalent in Asia and Africa, and esophageal adenocarcinoma (EAC), typically originating in the glandular epithelium of the lower esophagus and gastroesophageal junction, which has increased in incidence in Western countries over recent decades [[7], [8], [9], [10], [11]]. The two (02) major sub-types of EC differ in terms of their geographic prevalence and basic molecular and etiological features [[9], [10], [11], [12]]. ESCC is closely associated with tobacco use, excessive intake of alcohol, diets high in nitrosamines or pickled foods, poor nutrition, and environmental exposure to carcinogens such as polycyclic aromatic hydrocarbons [13,14]. By contrast, EAC is more commonly linked with chronic GERD (gastroesophageal reflux disease), Barrett's esophagus, obesity, and a diet high in fat and processed food [[15], [16], [17]].

The etiology of EC is influenced by the interplay between genetic predisposition and environmental exposures, instead of relying on a single determinant [6,11,15]. Environment linked factors that have been consistently associated with an increased risk of developing EC include the use of tobacco, excessive intake of alcohol, inadequate intake of fresh fruits and vegetables, nitrosamine-containing diets, and chronic irritation of the esophagus resulting from gastroesophageal reflux [13,15,16,18]. However, the level of risk associated with these exposures vary greatly among individuals, a variation due in great measures to genetic polymorphisms involved in modifying physiological responses to carcinogenic exposure [12,19].

Polymorphisms in an individual's genetic makeup, along with environmental and lifestyle factors, may influence the risk of developing EC [6,12,20]. Specific genetic polymorphisms in *TP53, XPC, ERCC1,* and *GSTP1*, which are associated with DNA repair, cell cycle regulation, and carcinogen metabolism, have been linked to differences in disease susceptibility and progression [21,22]. Genetic factors interact with environmental influences to produce notable variability in incidence rates, clinical features, and treatment outcomes across diverse populations affected with EC.

Variants in DNA repair genes like *XPC, ERCC1, XRCC1, XRCC2,* and *XRCC3* may decrease a cell's ability to repair DNA damage induced by environmental mutagens, thus hastening the accrual of mutations that promote neoplastic development [5,23,24]. Similarly, polymorphisms in alcohol-metabolizing genes, including *ALDH2* and *ADH1B*, determine the catabolism of ethanol and the clearance of acetaldehyde, a highly reactive and mutagenic by-product of alcohol metabolism [25,26]. These changes increase the susceptibility of carrying an alcohol-related cancer risk in genetically predisposed individuals. Together, these examples show how inherited genetic variation can either amplify or mitigate the effects of environmental carcinogens and supports the importance of integrating genomic profiling with environmental risk assessment to enhance the strategies of prevention and early detection in EC.

Single-nucleotide polymorphisms (SNPs) are the most common form of genetic variation in the human genome. It is a change occurring at a single nucleotide locus that can vary among individuals in a population [27]. In this vein, the subset of SNPs known as non-synonymous SNPs are especially significant because they appear in coding areas and result in the substitution of amino acid sites in proteins [28]. The substitutions may modify protein structure or function and, in some cases, may cause disease pathogenesis [29,30]. The main challenge is identifying which variants have functional impacts, considering the large data output from modern sequencing technologies [[27], [28], [29]]. Bioinformatics methods capable of distinguishing deleterious variants from neutrals are therefore necessary for prioritizing candidates for further *in vitro* or *in vivo* validation and possible therapeutics testing [28].

The present study aims to investigate how genetic variations predispose to EC by analyzing the nsSNPs in genes associated with this disease. First, genes associated with EC were aggregated from established genomic resources and the literature, and screened for rare-coding missense variants reported in large human datasets. A suite of well-validated computational tools was then used to assess which variants are most likely to disrupt protein stability, conformation, or function. To interpret these predictions in a biological context, evolutionary conservation of the affected residues was evaluated, and the ways in which each mutation alters physicochemical properties influencing folding and activity were examined. The effects of the most deleterious variants on protein architecture and folding have been further investigated by structural modeling and energy-based analysis. This work integrates these strands of evidence to identify a set of gene–protein alterations that may contribute to EC progression and might be a starting point for potential future therapeutic intervention.

## 2 Methodology

**Fig. 1:**
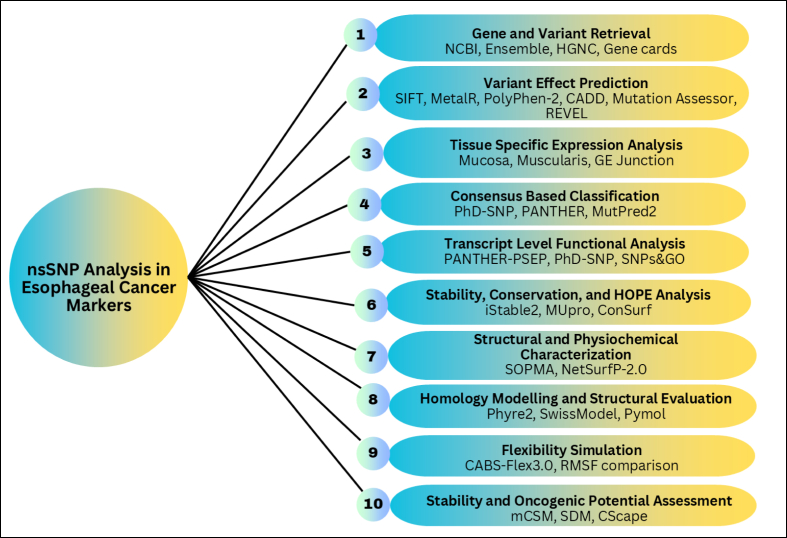
Stepwise outline of the study.

Fig. 1 sketch the methodological outline conducted throughout the study.

### 2.1 Gene retrieval, SNPs selection, and extraction of rare-coding, exonic mutations (nsSNPs)

Genes robustly associated with EC were identified through the NCBI Gene database (https://www.ncbi.nlm.nih.gov/genes). The search included both British and American spellings using the terms “*Oesophageal cancer*” and “*Esophageal cancer*.” The dataset included publications and records from January 1, 2014, through December 31, 2024. A total of 28 EC-related genes encoding 126 proteins, including their isoforms, were retrieved. For each gene, comprehensive genomic information was collected, including gene names, aliases, HGNC name/ID, NCBI accession number, ENSEMBL Gene ID, genomic coordinates, gene type, transcript and exon counts, variation data, protein names, amino acid lengths, UniProtKB identifiers, reference sequence status, and expression profiles. The required data were curated from NCBI (https://www.ncbi.nlm.nih.gov/gene), ENSEMBL (http://asia.ensembl.org/Homo_sapiens/Gene/Summary), the HUGO Gene Nomenclature Committee (HGNC; https://www.genenames.org/), and GeneCards (https://www.genecards.org/). The full workflow is presented in Fig. 1.

VEP was used to predict the functional consequence of the called variants, including SNPs, insertions, deletions, and SNVs, on the selected genes, transcripts, and protein-coding regions, including their regulatory elements [31]. The rare-coding variants were interrogated against the 1000 Genomes Project Database [32] and the Genome Aggregation Database (gnomAD) [33], using a MAF threshold of ≤0.01 (1%) as described by Asif et al [34]. For each SNP, the following information was noted: SNP ID, chromosomal position, nucleotide change, amino acid substitution, and transcript reference ID. Exonic variants were filtered using the ENSEMBL VEP interface; the results of interest were downloaded in CSV format for further analysis.

### 2.2 Identification of deleterious rare-coding SNPs using the Minor Allele Frequency (MAF) and DSFE prediction approach

To identify potentially harmful non-synonymous single nucleotide polymorphisms (nsSNPs) in the collected genomic dataset, several computational tools based on machine learning and deep learning algorithms were applied. Each tool contributes a unique perspective on how mutations might influence protein function and stability. All exonic variants of 28 EC-related genes were initially filtered based on MAF (minor allele frequency) and the cutoff set for MAF inclusion was ≤0.01 [34]. Afterwards, the main prediction tools used in this study are described below.

1. SIFT (Sorting Intolerant from Tolerant): SIFT (https://sift.bii.a-star.edu.sg/) helps distinguish between mutations that are likely to disrupt protein function and those that are harmless. It predicts how amino acid substitutions affect protein activity by generating a score between 0 and 1. Variants with lower scores are considered more likely to be deleterious [35].
2. PolyPhen-2 (Polymorphism Phenotyping v2): PolyPhen-2 (http://genetics.bwh.harvard.edu/pph2/dbsearch.shtml) evaluates the possible impact of amino acid changes on protein structure and function. Based on specificity and sensitivity parameters, mutations are classified as *probably damaging*, *possibly damaging*, or *benign.* These predictions were used for functional annotation of the analyzed genes [36].
3. CADD (Combined Annotation Dependent Depletion): CADD integrates multiple annotations to estimate the relative deleteriousness of single nucleotide variants and small insertions or deletions. Since higher CADD scores are strongly associated with greater functional impact and disease potential, a cutoff score of 20 or above was used to select nsSNPs with likely pathogenic effects [37].
4. REVEL (Rare Exome Variant Ensemble Learner): uses multiple predictive algorithms to classify missense mutations as pathogenic or benign. A score above 0.5 obtained from REVEL is observed for likely disease-causing variants, but below that threshold, it suggests benign variants. Previous evidence has suggested that the majority of known pathogenic mutations achieve scores greater than 0.5 and have hence established REVEL as a useful filter to prioritize variants [38].
5. MetaLR (Logistic Regression Meta-Predictor): MetaLR uses logistic regression to predict whether a single nucleotide variant is deleterious or tolerated. The scores range from 0 to 1, where a higher score indicates a higher probability of pathogenicity [28].
6. Mutation Assessor: This program predicts the functional consequences of amino acid substitutions based on evolutionary conservation patterns. Variants interpreted as high by Mutation Assessor are considered potentially damaging, and hence an elevated likelihood of disruption to normal protein activity [39].

Collectively, these prediction tools yield an integrated assessment of the deleterious potential of nsSNPs, thus enabling the fine-tuning of selecting mutations with the highest likelihood of functional and clinical significance. After identifying the rare-coding deleterious nsSNPs through the MAF and DSFE prediction approach, the next procedure was to filter out all VUS (variants of uncertain significance) variants [40]. VUS refers to genetic alterations whose functional impact is yet unclear and therefore should not be retained, for they will add ambiguity to further analyses. Since only high-confidence functionally relevant mutations were of interest in this study, all entries for VUS were filtered out systematically.

The filtering step incorporated aggregate pathogenicity scores from the previously used prediction tools, such as SIFT, PolyPhen-2, CADD, REVEL, MetaLR, and Mutation Assessor. All the variants that provided conflicting or borderline predictions from these algorithms were classified as VUS and removed. Again, population frequency data from the 1000 Genomes Project [32] and the gnomAD database [33] were revisited to confirm the rarity of the retained nsSNPs.

### 2.3 Pattern for tissues-specific expression of selected EC gene

To further illustrate how these selected genes express themselves in different parts of the esophagus, expression patterns were investigated using the GTEx database [41]. GTEx provides a rich resource of tissue-specific gene expression and associated regulatory variants. This study considered three regions of the esophagus: the gastroesophageal junction, mucosa, and muscularis. Analysis of these data allowed for the identification of genes most highly expressed within these tissues and a better understanding of how such expression might contribute to protein level changes relevant to EC biology.

### 2.4 Transcript sequence-based consensus classification of rare-coding, deleterious nsSNPs of EC biomarkers genes

To assess the functional impact of transcript-specific nonsynonymous SNPs, several independent prediction algorithms were used. Each tool considers amino acid substitutions from a different analytical point of view, which can allow more confident identification of mutations that might impair protein function. To unify the results in a more robust classification, the PredictSNP meta-analysis platform was employed, at http://loschmidt.chemi.muni.cz/predictsnp1/ [42]. This online tool integrates various high-performing predictors into a consensus score and overall prediction accuracy for each nsSNP. The PredictSNP integrates predictions from nine established tools: PhD-SNP, SNAP, PANTHER, PredictSNP, nsSNPAnalyzer, SIFT, PolyPhen-1, PolyPhen-2, and MAPP, which together predict the chances that any given variant is deleterious according to principles of evolutionary conservation, physicochemical properties, and protein structure changes. A joint approach has the advantage of reducing biases of the single prediction models and hence increases confidence in differentiating pathogenic mutations from neutral polymorphisms. Final classification results were used to prioritize the most functionally relevant nonsynonymous SNPs for further downstream analyses.

### 2.5 Transcript-level analysis of the functional impacts of nsSNPs in EC genes biomarkers using In Silico tools

All exonic missense variants identified in the earlier steps were examined further at the transcript level using a set of machine learning–based prediction tools. This analysis helped us assess whether each nsSNP was likely to be pathogenic. The following platforms were used:

1. PANTHER-PSEP

We used PANTHER-PSEP to estimate the evolutionary preservation of each amino acid position. The tool traces how long a residue has been conserved across ancestral lineages and reports preservation in millions of years. Positions maintained for long periods are more likely to be functionally important and, therefore, more sensitive to substitutions. Based on the preservation score, variants were categorized as probably damaging (over 450 million years), possibly damaging (200–450 million years), or probably benign (less than 200 million years) [43].

2. PhD-SNP

PhD-SNP applies a support vector machine to classify variants as disease-associated or neutral. The method is trained on the Swiss-Prot dataset and uses a 20-fold cross-validation strategy. Predictions were generated through the online server [44].

3. SNPs&GO

SNPs&GO predicts whether an amino acid change is disease related by combining sequence information, evolutionary features, and functional annotation derived from Gene Ontology terms. It also incorporates structure-based information when available. This integrated approach generally provides better discrimination between pathogenic and non-pathogenic variants [45].

4. MutPred2

MutPred2 evaluates the functional consequences of amino acid substitutions and assigns a probability score for pathogenicity. Scores of 0.5 or higher were considered indicative of a pathogenic effect, while lower values suggested a benign change [46].

### 2.6 Characterizing the effects of rare-coding nsSNPs on protein stability, evolutionary conservation and HOPE analysis using In Silico learning-based methods

To understand how rare-coding pathogenic nsSNPs might influence protein stability, structure, and evolutionary conservation, several supervised learning-based computational tools were employed. These tools combine statistical, machine learning, and evolutionary algorithms that predict the likely impact of amino acid substitutions on protein function *in silico*, without the use of laboratory experiments.

1. iStable v2.0: The iStable server (http://ncblab.nchu.edu.tw/iStable2/seqsubmit.html) makes use of a decision-tree–based learning method to classify whether a protein becomes stabilized or destabilized after a single amino acid substitution. It predicts the ΔΔG induced by mutations using the three residues adjacent to the mutated site on both the N and C-termini. Further, it calculates the solvent accessibility and secondary structure features to improve prediction ability. Integrating both classification and regression tree models, iStable scans the degree of structure and stability change in mutant proteins [47].
2. MUpro: MUpro (http://mupro.proteomics.ics.uci.edu/) combines support vector machines and neural network models for predicting protein stability changes upon point mutations. The major added advantage of this predictor is that it does not use the protein's 3D structure but instead relies on sequence-based features and physicochemical properties of amino acids to estimate whether a mutation increases or decreases stability. This makes MUpro very useful for studying the mutations in those proteins whose structures have not been resolved yet [48].
3. I-Mutant v3.0: I-Mutant v3.0 (http://gpcr2.biocomp.unibo.it/emidio/I-Mutant3.0/I-MutantSuiteHelp.html) predicts protein stability changes caused by single-point mutations under standard physiological conditions, pH 7.0 at 25 °C. The algorithm is based on a support vector machine that yields both classification and regression outputs in the form of ΔΔG values (kcal/mol), which reflect the magnitude and direction of the stability change. Predictions are separated into neutral mutations (−0.5 < ΔΔG <0.5), decreased stability (ΔΔG < −0.5), or increased stability (ΔΔG >0.5) classes, thereby simplifying the discrimination between mutations with minimal effects from those that strongly destabilize or stabilize a protein [49].
4. INPS-MD stands for the Impact of Non-synonymous Mutations on Protein Stability -Multi-Dimensional. INPS-MD (https://inpsmd.biocomp.unibo.it) predicts the impact of mutations on protein stability by considering both sequence-based and structure-based parameters. The model, implemented in the LIBSVM framework, combines linear and radial basis function kernels to predict changes in ΔΔG. Variants with ΔΔG >0 are considered stabilizing, while variants with ΔΔG <0 are destabilizing. In this regard, INPS-MD was specifically applied to all transcript-level nsSNPs, identified in this work, which provided a quantitative measure of their potential impact on protein folding and thermodynamic stability [50].
5. DDGun: https://folding.biofold.org/ddgun/DDGun predicts the impact of single or multiple amino acid substitutions on protein stability. It integrates sequence features and evolutionary information, including the BLOSUM62 substitution matrix into its scoring scheme. Instead of returning a specific BLOSUM score, DDGun uses the substitution values in its model to calculate the predicted ΔΔG changes, which gives an indication if the mutation would stabilize or destabilize the protein structure [51].
6. ConSurf: The evolutionary importance of every amino acid residue was analyzed through the ConSurf web server at http://consurf.tau.ac.il. ConSurf uses a Bayesian statistical algorithm in order to estimate the evolutionary conservation scores across multiple sequence alignments. The highly conserved residues, which have scores ranging from 7 to 9, are considered critical to both structural and functional integrity and thus confer lesser tolerance to mutations [52].
7. HOPE server: To further interpret the structural and functional impacts of amino acid substitutions, all mutations were analyzed with the HOPE server [Have (y)Our Protein Explained; http://www.cmbi.ru.nl/hope/input/]. This tool gives an overview of how a given change may affect a protein by studying size, charge, hydrophobicity, and other physicochemical properties. It investigates how a mutation will affect a local structural environment, folding of a protein, and its possible functional implications. The reports generated provided insight into whether each substitution could disrupt the protein or distort key structural features of the protein [53].

### 2.7 Assessment of the impacts of the transcript-level structural changes of rare-coding, deleterious nsSNPs on protein conformations, energetics, and dynamics

The following are various computational tools employed in studying structural parameters, including secondary structure organization, solvent accessibility, torsion angles, energetic potentials, and overall conformational stability, in order to estimate the impact of rare-coding nsSNPs on protein structure and dynamics. The SOPMA tool was used to predict the protein secondary structural features, including α-helices, β-sheets, turns, and coils. SOPMA generates a consensus secondary structure profile by incorporating five different prediction methods using neural networks and probabilistic approaches. SOPMA analyses have illuminated the structural and evolutionary contexts of studied nsSNPs, providing a clear view on how these variations may affect protein function and stability [54].

A server developed at DTU, NetSurfP-2.0 (https://services.healthtech.dtu.dk/services/NetSurfP-2.0/), was used for the prediction of secondary structure composition, surface accessibility, and percentage disordered residues for each of these proteins. This server also computes the backbone torsional angles, namely phi (φ) and psi (ψ), providing information on local folding patterns and conformational flexibility of the protein chain [55].

### 2.8 Transcript-level protein homology modelling and evaluation of structural modifications impact on proteins’ functions

The three-dimensional (3D) structures of protein variants derived from each transcript sequence were predicted using the Phyre2 (Protein Homology/analogY Recognition Engine v2.2) and SWISS-MODEL web-based platforms.

Phyre2 v2.2 (https://www.sbg.bio.ic.ac.uk/∼phyre2/html/page.cgi?id=index) applies homology modeling principles to predict protein folding and spatial arrangement by comparing the target sequence to known protein templates with experimentally resolved structures [56]. The residue specific modified 3D model (mutant types) was made from PyMOL 3.1 (https://www.pymol.org/) molecular visualization system on an open-source foundation, maintained and distributed by Schrödinger [57]. Through the use of sequence alignment and profile–profile matching based on Hidden Markov Models (HMMs), Phyre2 identifies distant structural homologs and generates accurate models even when sequence identity is low. The tool also provides confidence scores, alignment coverage, and secondary structure predictions that help assess the quality of the resulting model.

In addition to Phyre2, SWISS-MODEL (https://swissmodel.expasy.org/) was employed to generate and validate homology-based three-dimensional structures. This automated server, hosted on the ExPASy bioinformatics resource portal, uses a template-based modeling approach in which each target sequence is aligned with the most appropriate structural template available in the Protein Data Bank (PDB). The modeling workflow has four principal steps: template identification, target–template alignment, model construction, and structural quality evaluation. SWISS-MODEL predicts Global Model Quality Estimation (GMQE) and Qualitative Model Energy Analysis (QMEAN) scores for each predicted model regarding the reliability and stability of the model. These metrics provide insight into the structural accuracy and energetic plausibility of the protein conformations that are produced [58].

Subsequently, possible changes in conformation that amino acid substitution can induce were investigated through the integrated web server DynaMut, https://biosig.lab.uq.edu.au/dynamut/, combining normal mode analysis with graph-based signatures to assess protein flexibility and stability. DynaMut predicts the effects of specific residue mutations on vibrational entropy and overall protein dynamics to help determine whether introduced structural changes by rare-coding nsSNPs enhance or destabilize the native conformation [59]. Overall, from transcript level, these various modeling and computational analyses provide a full understanding of how variants affect protein folding, energetic potential, and stability of conformation, and thus provide structural insights into the possible functional consequences of rare-coding nonsynonymous substitutions.

### 2.9 Wild type and mutant type model structure comparison for protein flexibility analysis

To assess the impact of transcript-level nsSNPs on the tertiary structure and conformational dynamics for each protein, wild-type and variant models were analyzed using the CABS-Flex 3.0 web server, https://lcbio.pl/cabsflex3/. This web server enables a highly efficient simulation of protein flexibility by means of a coarse-grained model and delivers insights into the mobility of residues and overall structural behavior. For all simulations, the total number of cycles and the interval between trajectory frames were set to 50, while all other parameters remained at their default values to provide a uniform conditions basis among the different models. The flexibility patterns generated from CABS-Flex 3.0 have been further analyzed using Root Mean Square Fluctuation (RMSF) analysis, a measure of per-residue movement within a protein structure. RMSF for wild-type and mutant proteins was visualized with the help of the Pandas library in Python on the Google Colab platform to clearly compare the flexibility profile and assist in determining residues where amino acid substitutions might have had an effect on stability or dynamic behavior [60].

### 2.10 Computational prediction of protein stability function and oncogenic potential effects of rare-coding, deletrious nsSNPs of EC-biomarker

To further understand how these identified rare-coding missense variants may influence protein behavior in esophageal cancer markers, a set of structure-based prediction tools was engaged to assess protein stability, folding, and even potential oncogenic effects. mCSM (http://structure.bioc.cam.ac.uk/mcsm) predicts the effects of amino acid substitutions by describing the local atomic environments using graph-based signatures that capture atomic arrangement around the mutation site, thus enabling the prediction of changes in the stability of folded proteins [61]. SDM (Site Directed Mutator; http://www-cryst.bioc.cam.ac.uk/∼sdm/sdm.php) predicts the effects of mutations from substitution probability tables that have been derived from protein families of known structure, therefore quantifying how a given residue replacement may affect stability within its native structural context [62]. DUET, on the other hand, merges both mCSM and SDM into a single consensus model using a support vector machine to combine the strengths of both methods, hence yielding more reliable stability predictions for single-point mutations [63]. DynaMut2 (https://biosig.lab.uq.edu.au/dynamut2/) was also engaged for addressing the impact of mutations on both stability and conformational flexibility by normal mode analysis assessing the change in vibrational entropy and overall protein dynamics upon residue substitution [64].

The CUPSAT (Cologne University Protein Stability Analysis Tool; https://cupsat.brenda-enzymes.org/) server was included for the further assessment of the thermodynamical consequences of each mutation. CUPSAT predicts the change in free energy (ΔΔG) between the wild-type and mutant structures by analyzing torsion angle preferences and residue-specific structural environments. This helped us determine whether each variant was likely to stabilize or destabilize the protein [65].

To assess whether any of the rare-coding deleterious nsSNPs might contribute to oncogenic behavior, we used CScape (http://CScape.biocompute.org.uk/.). This tool differentiates potential cancer “driver” mutations from neutral “passenger” variants by assigning a probability score to each change. Scores approaching 1 indicate a higher likelihood that the variant contributes to cancer development. CScape has been shown to perform well across both coding and non-coding regions, making it a suitable choice for evaluating the oncogenic potential of the mutations in our dataset [66].

## 3 Results

### 3.1 EC-related genes and protein biomarkers

A comprehensive list of esophageal cancer-associated biomarker genes was retrieved from the NCBI database, covering studies published between January 2014 and December 2024 (S1 Table). The list provides a compilation of validated and newly known disease-causing and molecular signaling genes in relation to EC, wherein each entry provides detailed genomic and proteomic information, such as official gene symbol, chromosomal location, RefSeq ID, transcript variant, protein length, and UniProt accession number, as shown in S1 Table.

The analysis shows that most of the genes implicated in EC are protein coding, highlighting their functional relevance to the processes of tumorigenesis and tumor progression. Chromosomal mapping reveals a wide genomic distribution with clustering on chromosomes 1, 7, 12, 17, and 20-regions commonly reported to host oncogenic drivers or tumor suppressors involved in epithelial malignancies (see S1 Table). S1 Table also highlights significant variation in exon number and transcript isoform number, suggesting that alternative splicing and post-transcriptional regulation might contribute further to the molecular heterogeneity seen in esophageal cancer. For example, *ELMO2* (20q13.12) and *RNF187* (1q42.13) have multiple transcript variants with a high degree of exonic variation, reflecting activity in dynamic signaling networks and transcriptional regulation. Similarly, *INPP5J* (22q12.2) exhibits extensive genetic variation, with over 57,000 variants, suggesting genomic instability and the potential for this gene to be used as a diagnostic or therapeutic target. Most of the EC gene biomarkers mentioned in this table have their associated information curated and annotated as ‘reviewed’ or ‘validated’ in RefSeq, hence giving credence to the curated dataset. Various details on protein annotation range from ∼200 to more than 1000 amino acids, representing a wide array of structural and functional features. In addition, corresponding UniProt entries support experimentally provided evidence for protein identity and functional domains related to carcinogenic pathways (S1 Table).

**Fig. 2:**
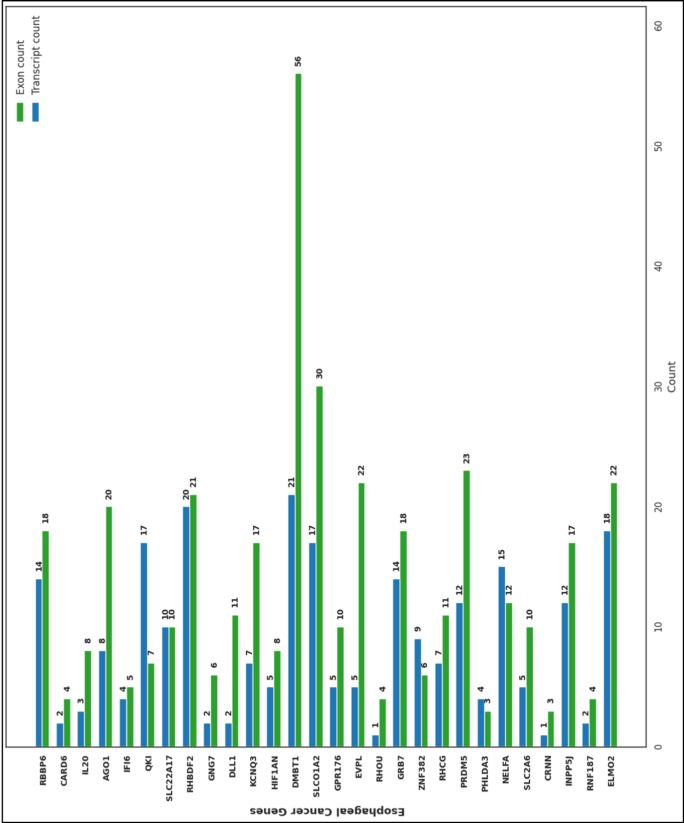
Exon and transcript distribution among EC-related genes biomarkers.

Among the studied genes, *DMBT1* contains the highest number of exons (56) and a relatively high number of transcripts (21), indicating a high alternative splicing activity with potentially high functional complexity of the encoded protein. *SLC10A2* and *PRDM5* also contain higher exon counts, with 30 and 23 exons, respectively. *EVPL* has the highest number of transcripts, at 22, despite fewer numbers of exons, which indicates possible regulatory variability at the transcript level (Fig. 2). In contrast, many genes, including *RHOU*, *CRNN*, and *CARD6*, have low numbers of exons and transcripts, suggesting little structural variation. Many genes, including *RBBP6*, *RHCG*, *IFI6*, and *GRB7*, have intermediate exon–transcript ratios and represent a balance between levels of transcriptional diversity (Fig. 2).

### 3.2 Mutational estimates of the EC-associated gene biomarkers

**Fig. 3:**
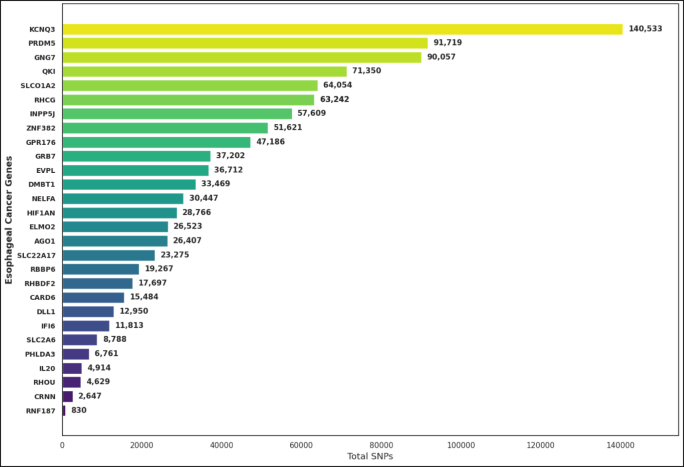
Total number of genetic variations/mutations present across EC–related genes biomarkers.

A total of 28 EC–associated genes were analyzed for nucleotide polymorphisms (SNPs) retrieved from the NCBI database from January 2014 to December 2024. Generally, there was a substantial variation in the whole SNP distribution pattern among these EC-genes, reflecting extensive genomic diversity within this set of EC candidate biomarkers (Fig. 3). Among these genes, *KCNQ3* harbored the highest number of SNPs (140,533), followed by *PRDM5* (91,719), *GNG7* (90,057), and *QKI* (71,350). The genes discussed herein are known for their fundamental contribution in modulating cellular signaling, differentiation, and transcription. It is envisaged that their high frequencies of mutation will contribute to esophageal tumorigenesis by modulating protein functions or disrupting key signaling pathways. By contrast, variants were fewer in number in *RNF187* (830 SNPs), *CRNN* (2647 SNPs), and *RHOU* (4629 SNPs), thus showing higher genomic stability and less mutational tolerance at these loci (Fig. 3).

*The SLC01A2, RHCG,* and *INPP5J* genes showed intermediate SNP frequencies between 57,000 and 64,000 variants per gene. These genes are respectively responsible for ion transport and phosphoinositide signaling, both of which are involved in pathways in epithelial homeostasis or tumorigenicity. Membrane transport genes, as well as those related to transcriptional regulation and cellular communication, tended to possess higher sequence variations, consistent with the pattern of variation shown in Fig. 3.

**Fig. 4:**
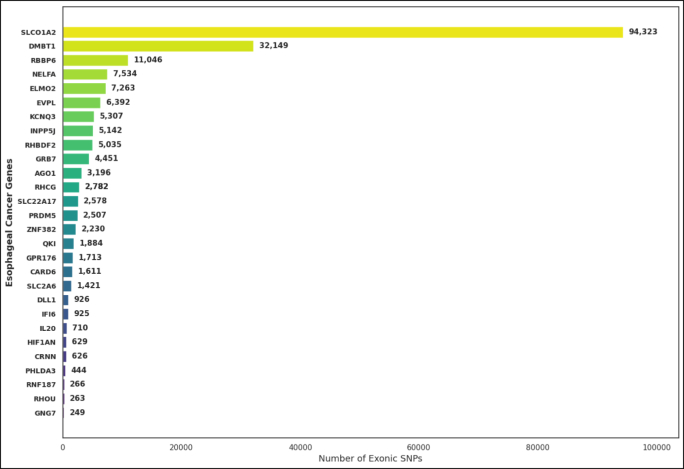
Distribution of exonic mutations across EC–related genes biomarkers.

There was a great variation in the distribution of exonic SNPs among these 28 EC-associated genes throughout the dataset, reflecting pronounced differences in alterations to the coding sequence, as shown in Fig. 3. Conspicuously, *SLCO1A2* had the highest number of exonic variants (94,323), followed by *DMBT1* (32,149) and *RBBP6* (11,046). These genes participate in molecular transport, regulation of immune responses, and control of the cell cycle, and disruption of these functions may engender carcinogenic transformation, as shown in Fig. 4.

We identified moderate SNP frequencies for *NELFA* (7,534), *ELMO2* (7,263), *EVPL* (6,392), *KCNQ3* (5307), and *INPP5J* (5,142), suggesting that coding variants in these genes might impact transcriptional regulation and epithelial integrity. Thus, the majority of genes, such as *PRDM5*, *ZNF382*, and *GPR176*, had a moderate number of exonic variants ranging between 1700 and 2,500, which postulates functional relevance at a lower mutation density compared to transporter and scaffolding proteins, as illustrated in Fig. 4.

In contrast, a subset of genes, including *RNF187* (266 SNPs), *RHOU* (263 SNPs), and *GNG7* (249 SNPs), had relatively few exonic mutations, suggesting either stronger evolutionary conservation or lesser susceptibility to changes within the coding region. The fewer SNPs in genes like *PHLDA3*, *CRNN*, and *HIF1AN* indicate limited sequence tolerance, possibly due to their crucial functions in cellular homeostasis maintenance (see Fig. 4). This pattern of exonic SNPs reflects those genes related to membrane transport and structural stability contain the highest number of coding variants, whereas genes involved in regulatory and metabolic control are more conserved. Together, these findings reflect the complex mutational landscape of EC, in which both highly variable and highly conserved genes may contribute concurrently to disease susceptibility and progression.

### 3.3 Transcript-level computational screening and functional prediction of deleterious nsSNPs in EC genes biomarker

**Fig. 5:**
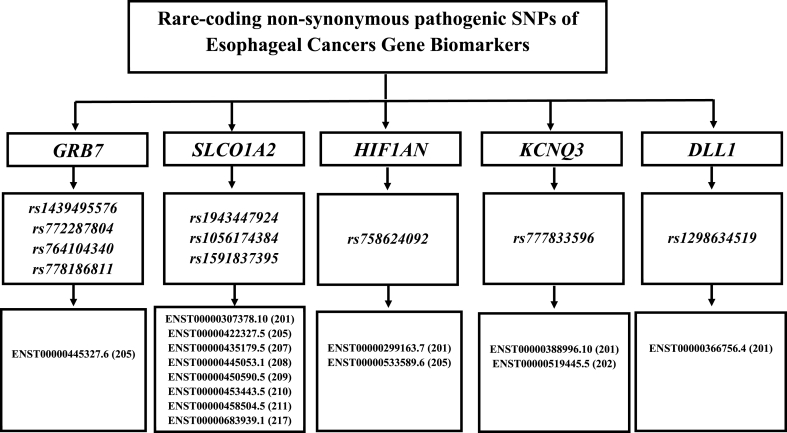
Distribution of rare-coding deleterious nsSNPs across transcript variants of EC biomar**ker genes.** The figure illustrates the distribution of rare-coding nsSNPs identified within transcript sequences of EC-associated genes. Each filtered gene is annotated to its corresponding ENSEMBL transcript version, with a focus on the localization of pathogenic variants at the level of transcripts. The pertinent dataset was assembled through a multi-step annotation and filtering workflow, prioritizing functionally relevant and potentially deleterious SNPs based on the results of downstream predictive analysis.

From an initial pool of 28 EC-associated genes retrieved from the NCBI database covering the period from January 2014 to December 2024, downstream functional effect (DSFE) analysis was conducted to identify rare-coding variants with potential pathogenic effects. After filtering based on minor allele frequency across population databases and applying multiple pathogenicity prediction tools, five genes carrying deleterious nonsynonymous substitutions were shortlisted: *GRB7*, *SLCO1A2*, *HIF1AN*, *KCNQ3*, and *DLL1* (see Fig. 5). The variants and associated evidence are summarized in Table 1 and S2 Table.

**Table 1:** Extraction of rare-coding deleterious nsSNPs through downstream functional effects (DSFE) predictions on genomic variants of selected EC biomarkers genes.

Gene	Variant ID	Position	Transcript ID	Evidence	Consequence Type	SIFT class	Polyphen class	CADD class	REVEL class	MetaLR class	Mutation assessor class
*GRB7*	rs1439495576	W454C	205	Frequency ∼ gnomAD	missense variant	deleterious	probably damaging	likely deleterious	likely disease causing	damaging	high
*GRB7*	rs772287804	G457W	205	Frequency ∼ ExAC ∼ TOPMed ∼ gnomAD	missense variant	deleterious	probably damaging	likely deleterious	likely disease causing	damaging	high
*GRB7*	rs764104340	R461H	205	Frequency∼1000Genomes ∼ ExAC ∼ TOPMed ∼ gnomAD	missense variant	deleterious	probably damaging	likely deleterious	likely disease causing	damaging	high
*GRB7*	rs764104340	R461L	205	Frequency∼1000Genomes ∼ ExAC ∼ TOPMed ∼ gnomAD	missense variant	deleterious	probably damaging	likely deleterious	likely disease causing	damaging	high
*GRB7*	rs778186811	R481Q	205	Frequency ∼ ExAC ∼ TOPMed ∼ gnomAD	missense variant	deleterious	probably damaging	likely deleterious	likely disease causing	damaging	high
*SLCO1A2*	rs1943447924	D572Y	201	Frequency	missense variant	deleterious	probably damaging	likely deleterious	likely disease causing	damaging	high
*SLCO1A2*	rs1056174384	G369R	201	Frequency ∼ TOPMed ∼ gnomAD	missense variant	deleterious	probably damaging	likely deleterious	likely disease causing	damaging	high
*SLCO1A2*	rs1591837395	G68R	201	Frequency	missense variant ∼ splice region variant	deleterious	probably damaging	likely deleterious	likely disease causing	damaging	high
*SLCO1A2*	rs1591837395	G68R	205	Frequency	missense variant ∼ splice region variant	deleterious	probably damaging	likely deleterious	likely disease causing	damaging	high
*SLCO1A2*	rs1591837395	G68R	207	Frequency	missense variant ∼ splice region variant	deleterious	probably damaging	likely deleterious	likely disease causing	damaging	high
*SLCO1A2*	rs1591837395	G66R	208	Frequency	missense variant ∼ splice region variant	deleterious	probably damaging	likely deleterious	likely disease causing	damaging	high
*SLCO1A2*	rs1591837395	G68R	209	Frequency	missense variant ∼ splice region variant	deleterious	probably damaging	likely deleterious	likely disease causing	damaging	high
*SLCO1A2*	rs1591837395	G68R	210	Frequency	missense variant ∼ splice region variant	deleterious	probably damaging	likely deleterious	likely disease causing	damaging	high
*SLCO1A2*	rs1943447924	D440Y	211	Frequency	missense variant	deleterious	probably damaging	likely deleterious	likely disease causing	damaging	high
*SLCO1A2*	rs1056174384	G237R	211	Frequency ∼ TOPMed ∼ gnomAD	missense variant	deleterious	probably damaging	likely deleterious	likely disease causing	damaging	high
*SLCO1A2*	rs1943447924	D572Y	217	Frequency	missense variant	deleterious	probably damaging	likely deleterious	likely disease causing	damaging	high
*SLCO1A2*	rs1056174384	G369R	217	Frequency ∼ TOPMed ∼ gnomAD	missense variant	deleterious	probably damaging	likely deleterious	likely disease causing	damaging	high
*SLCO1A2*	rs1591837395	G68R	217	Frequency	missense variant ∼ splice region variant	deleterious	probably damaging	likely deleterious	likely disease causing	damaging	high
*HIF1AN*	rs758624092	D201H	201	Frequency	missense variant	deleterious	probably damaging	likely deleterious	likely disease causing	damaging	high
*HIF1AN*	rs758624092	D94H	205	Frequency	missense variant	deleterious	probably damaging	likely deleterious	likely disease causing	damaging	high
*KCNQ3*	rs777833596	E170G	201	Frequency ∼ Phenotype_or_Disease ∼ ExAC ∼ gnomAD	missense variant and pathogenic	deleterious	probably damaging	likely deleterious	likely disease causing	damaging	high
*KCNQ3*	rs777833596	E170G	202	Frequency ∼ Phenotype_or_Disease ∼ ExAC ∼ gnomAD	missense variant and pathogenic	deleterious	probably damaging	likely deleterious	likely disease causing	damaging	high
*DLL1*	rs1298634519	Y389 N	201	Frequency ∼ gnomAD	missense variant	deleterious	probably damaging	likely deleterious	likely disease causing	damaging	high

Each selected variant underwent functional cross-validation using SIFT, PolyPhen-2, CADD, REVEL, MetaLR, and Mutation Assessor. All shortlisted nsSNPs consistently showed damaging or deleterious classifications across these tools, with high confidence scores indicating their potential involvement in functional disruption. Most variants were classified as “deleterious” by SIFT and “probably damaging” by PolyPhen-2, while CADD, REVEL, and MetaLR predicted a strong disease-causing potential. The Mutation Assessor scores were invariably suggestive of a high functional impact.

Four rare coding missense variants in the *GRB7* gene, rs1439495576, rs772287804, rs764104340 and rs778186811, all lying in the same transcript, ENST00000445327.6, thus point towards a localized region of functional sensitivity. Multiple deleterious transcript-specific variants have also been identified in *SLCO1A2*, including G68R, G369R, D440Y, and D572Y, several of which appear repeatedly across different transcript isoforms. Their consistent classification as damaging strengthens the possibility that these substitutions affect transporter-related functions relevant to cancer.

The *HIF1AN* variant D201H demonstrated strong damaging predictions, suggesting potential interference with hypoxia-related regulatory processes in the body. Similarly, the *KCNQ3* variant E170G was supported by both population-level and phenotype-based evidence and was classified as deleterious by all predictive tools, indicating possible alterations in channel function. In the *DLL1* gene, Y389 N has been identified as a rare-coding disease-causing substitution.

### 3.4 Tissue-specific expression patterns of selected EC genes biomarkers

To further explain the behavior of the filtered gene set within tissues pertinent to EC, we assayed their expression patterns across three esophageal sites using GTEx data: the gastroesophageal junction, esophageal mucosa, and muscularis layer. The goal was to determine if any of the selected markers exhibit tissue-specific activity that might relate to the biology of the disease.

Although most genes showed measurable expression in all three tissues, their expression levels varied widely. *GRB7* exhibited a strong site-specific pattern with high median expression (TPM ≈ 62) in the mucosa, while it remained low in both the gastroesophageal junction and the muscularis. Such striking contrast indicates that its activity may be highly epithelial rather than in deeper structural tissues (S3 Table).

Conversely, *SLCO1A2* showed very low expression in all esophageal tissues, with a median TPM less than 0.01, and its detectable expression seems not adequate for an important transcriptional function in the normal esophagus biology (S3 Table).

*HIF1AN* presented a more balanced expression profile, with a moderate expression across the three tissue types, while expression levels ranged from about 9.5 TPM in the mucosa to more than 13 TPM in both the gastroesophageal junction and muscularis, suggesting that this gene is also widely active in the esophagus and not restricted to any particular area of this organ (S3 Table). The expression of *KCNQ3* was overall low, but constant across the tissues. Values remained below 0.5 TPM, with only small differences between the sites. This suggests that although *KCNQ3* is present, it may not be a major contributor to the baseline transcriptional activity in the esophagus (S3 Table).

*DLL1* showed relatively high expression, especially in the mucosa, where the median expression level reached nearly 29 TPM. Expression in the gastroesophageal junction and muscularis was lesser. The raised mucosal (tissue) expression pattern aligns with the particular gene's function/role in Notch signaling, that affects epithelial differentiation (S3 Table).

Comparatively, the mucosal layer demonstrated the highest expression of several genes, most clearly for *GRB7* and *DLL1*. This pattern supports the idea that many of the filtered markers have stronger functional relevance in epithelial compartments, which are also the primary sites of malignant transformation in esophageal cancer (S3 Table).

### 3.5 Consensus-based prediction of rare-coding deleterious nsSNPs in EC biomarker genes

To identify potentially pathogenic amino acid substitutions among the shortlisted biomarker genes, a consensus classifier approach was applied using the PredictSNP platform, which integrates seven independent algorithms (MAPP, PhD-SNP, PolyPhen-1, PolyPhen-2, SIFT, and SNAP). This multi-model analysis validated and strengthened the confidence in functional impact predictions by combining different computational algorithms of protein sequence conservation and structural context.

Among the five selected EC–associated genes (*GRB7*, *SLCO1A2*, *HIF1AN*, *KCNQ3*, and *DLL1*), several rare-coding nsSNPs showed consistently high deleterious prediction scores. Within *GRB7*, four key variants, W454C, G457W, R461 H/L, and R481Q, showed accuracy percentages ranging from 68% to 88% across classifiers, with R461H and R461L yielding particularly high confidence (85% by SNAP and 82% by PhD-SNP), suggesting strong pathogenic potential (Table 2).

**Table 2:** Percent estimation of expected accuracy of rare-coding deleterious nsSNPs of selected EC biomarker genes through consensus classifier prediction approach.

Gene	Variant ID	Position	Transcript ID	Predict SNP (%)	MAPP (%)	PhD-SNP (%)	Polyphen-1 (%)	Polyphen-2 (%)	SIFT (%)	SNAP (%)
*GRB7*	rs1439495576	W454C	205	87^a^	43^a^	88^a^	74^a^	68^a^	79^a^	81^a^
*GRB7*	rs772287804	G457W	205	76^a^	64^b^	82^a^	74^a^	81^a^	79^a^	81^a^
*GRB7*	rs764104340	R461H	205	72^a^	75^b^	82^a^	74^a^	81^a^	79^a^	85^a^
*GRB7*	rs764104340	R461L	205	76^a^	70^b^	77^a^	74^a^	81^a^	79^a^	85^a^
*GRB7*	rs778186811	R481Q	205	72^a^	77^b^	61^a^	59^a^	81^a^	79^a^	81^a^
*SLCO1A2*	rs1943447924	D572Y	201	87^a^	76^a^	88^a^	74^a^	-	79^a^	87^a^
*SLCO1A2*	rs1056174384	G369R	201	87^a^	88^a^	86^a^	59^a^	81^a^	79^a^	85^a^
*SLCO1A2*	rs1591837395	G68R	201	87^a^	88^a^	88^a^	59^a^	-	79^a^	85^a^
*SLCO1A2*	rs1591837395	G68R	205	87^a^	88^a^	88^a^	59^a^	81^a^	79^a^	85^a^
*SLCO1A2*	rs1591837395	G68R	207	-	88^a^	88^a^	59^a^	81^a^	79^a^	89^a^
*SLCO1A2*	rs1591837395	G66R	208	87^a^	-	88^a^	74^a^	81^a^	79^a^	85^a^
*SLCO1A2*	rs1591837395	G68R	209	87^a^	88^a^	88^a^	59^a^	81^a^	79^a^	89^a^
*SLCO1A2*	rs1591837395	G68R	210	87^a^	88^a^	88^a^	59^a^	81^a^	79^a^	89^a^
*SLCO1A2*	rs1943447924	D440Y	211	-	77^a^	88^a^	74^a^	81^a^	79^a^	87^a^
*SLCO1A2*	rs1056174384	G237R	211	-	88^a^	86^a^	59^a^	81^a^	79^a^	89^a^
*SLCO1A2*	rs1943447924	D572Y	217	87^a^	76^a^	88^a^	74^a^	81^a^	79^a^	87^a^
*SLCO1A2*	rs1056174384	G369R	217	87^a^	88^a^	86^a^	59^a^	81^a^	79^a^	85^a^
*SLCO1A2*	rs1591837395	G68R	217	87^a^	88^a^	88^a^	59^a^	81^a^	79^a^	85^a^
*HIF1AN*	rs758624092	D201H	201	87^a^	77^a^	88^a^	74^a^	81^a^	79^a^	87^a^
*HIF1AN*	rs758624092	D94H	205	76^a^	77^a^	88^a^	74^a^	81^a^	68^b^	87^a^
*KCNQ3*	rs777833596	E170G	201	87^a^	86^a^	82^a^	59^a^	41^a^	79^a^	85^a^
*KCNQ3*	rs777833596	E170G	202	87^a^	86^a^	82^a^	74^a^	81^a^	79^a^	81^a^
*DLL1*	rs1298634519	Y389 N	201	61^a^	56^a^	55^b^	59^a^	41^a^	79^a^	50^b^

a Damaging or deleterious.

b Neutral; ‘-’ Not computed by consensus classifier.

For *SLCO1A2*, multiple transcript substitution events showed predictive scores (G68R, G369R, D440Y, and D572Y) constantly between 85% and 89% across different tools (Table 2). This indicates a strong consensus regarding deleterious changes. Similar results were observed for the *HIF1AN* variants (D201H and D94H), which reached 87% in most algorithms, suggesting potential interruption of pathways for oxygen sensing or transcriptional regulation (Table 2). The *KCNQ3* variant E170G exhibited moderate-to-high deleterious probabilities, ranging from 82 to 87%, suggesting possible alteration of ion channel function related to epithelial cell homeostasis (Table 2). In contrast, the *DLL1* Y389 N variant demonstrated lower predictive scores, 50-61%, and was consistently classified as neutral by several tools, indicating reduced functional consequences (Table 2). In general, the consensus prediction approach efficiently filtered disease-associated variants, identifying rare-coding nsSNPs with high pathogenic potential across multiple models. These variants are promising candidates for further structural modeling and molecular validation of EC pathogenesis.

### 3.6 Sequence-based analysis of rare-coding deleterious nsSNPs in EC biomarker genes

Sequence-level pathogenicity assessment of the filtered rare-coding deleterious nsSNPs was performed using five independent predictive models: PANTHER-PSEP, PANTHER, PhD-SNP, SNP&GO, and MutPred2. The basic purpose of this step was to evaluate the probability and reliability of functional impact across the filtered five EC biomarker genes. The integration of these tools enabled the cross-validation of predicted deleteriousness by combining evolutionary conservation, sequence homology, and structural property-based scores.

In *GRB7*, the variants W454C, G457W, R461 H/L, and R481Q consistently showed “probably damaging” outcomes in PANTHER-PSEP, with high reliability indices across PhD-SNP and SNP&GO platforms. W454C and G457W were predicted to be disease-associated, with probability values exceeding 0.8 in both PhD-SNP and SNP&GO, whereas MutPred2 highlighted potential alterations in the ordered interface regions. The mutation R461L appeared most significant among those because of the predicted loop gain based on MutPred2 score (0.729), indicating a potential conformational change affecting *GRB7* protein–protein interactions (Table 3).

**Table 3:** Transcript sequence-based integrated functional impact prediction of rare-coding, deleterious nsSNPs in EC biomarker variants.

Gene	Variant ID	Position	Transcript ID	PANTHER-PSEP	PANTHER (Probability/RI/Prediction)	PhD-SNP (Probability/RI/Prediction)	SNP&GO (Probability/RI/Prediction)	MutPred2
*GRB7*	rs1439495576	W454C	205	1037/0.85 (probably damaging)	0.664/3/Disease	0.844/7/Disease	0.674/3/Disease	0.752 (Altered Ordered interface)
*GRB7*	rs772287804	G457W	205	842/0.78 (probably damaging)	0.807/6/Disease	0.772/5/Disease	0.551/1/Disease	0.72 (Altered Ordered interface)
*GRB7*	rs764104340	R461H	205	1036/0.85 (probably damaging)	0.419/2/Neutral	0.780/6/Disease	0.472/1/Neutral	0.542
*GRB7*	rs764104340	R461L	205	1036/0.85 (probably damaging)	0.319/4/Neutral	0.869/7/Disease	0.602/2/Disease	0.729 (Gain of Loop)
*GRB7*	rs778186811	R481Q	205	1036/0.85 (probably damaging)	0.660/3/Disease	0.818/6/Disease	0.669/3/Disease	0.708 (Loss of Pyrrolidone carboxylic acid at Q484)
*SLCO1A2*	rs1943447924	D572Y	201	1036/0.85 (Probably damaging)	-	0.921/8/Disease	0.7/4/Disease	0.91
*SLCO1A2*	rs1056174384	G369R	201	1036/0.85 (Probably damaging)	0.832/7/Disease	0.963/9/Disease	0.867/7/Disease	0.907
*SLCO1A2*	rs1591837395	G68R	201	910/0.85 (Probably damaging)	0.998/10/Disease	0.968/9/Disease	0.903/7/Disease	0.828
*SLCO1A2*	rs1591837395	G68R	205	910/0.85 (Probably damaging)	-	0.915/8/Disease	0.670/3/Disease	0.856
*SLCO1A2*	rs1591837395	G68R	207	910/0.85 (Probably damaging)	-	0.915/8/Disease	0.701/4/Disease	0.87
*SLCO1A2*	rs1591837395	G66R	208	910/0.85 (Probably damaging)	-	0.748/5/Disease	0.380/2/Neutral	0.938
*SLCO1A2*	rs1591837395	G68R	209	910/0.85 (Probably damaging)	-	0.915/8/Disease	0.703/4/Disease	0.865
*SLCO1A2*	rs1591837395	G68R	210	910/0.85 (Probably damaging)	-	0.913/8/Disease	0.668/3/Disease	0.838
*SLCO1A2*	rs1943447924	D440Y	211	1036/0.85 (Probably damaging)	0.998/10/Disease	0.968/9/Disease	0.902/8/Disease	0.911
*SLCO1A2*	rs1056174384	G237R	211	1036/0.85 (Probably damaging)	0.832/7/Disease	0.963/9/Disease	0.868/7/Disease	0.934
*SLCO1A2*	rs1943447924	D572Y	217	1036/0.85 (Probably damaging)	0.998/10/Disease	0.968/9/Disease	0.903/8/Disease	0.91
*SLCO1A2*	rs1056174384	G369R	217	1036/0.85 (Probably damaging)	0.832/7/Disease	0.963/9/Disease	0.867/7/Disease	0.907
*SLCO1A2*	rs1591837395	G68R	217	910/0.85 (Probably damaging)	-	0.921/8/Disease	0.7/4/Disease	0.828
*HIF1AN*	rs758624092	D201H	201	1237/0.85 (Probably damaging)	0.632/3/Disease	0.878/8/Disease	0.586/2/Disease	0.948
*HIF1AN*	rs758624092	D94H	205	1237/0.85 (Probably damaging)	0.634/3/Disease	0.871/7/Disease	0.574/1/Disease	0.833
*KCNQ3*	rs777833596	E170G	201	1037/0.85 (Probably damaging)	0.927/9/Disease	0.942/9/Disease	0.832/7/Disease	0.912
*KCNQ3*	rs777833596	E170G	202	1037/0.85 (Probably damaging)	0.927/9/Disease	0.941/9/Disease	0.832/7/Disease	0.882
*DLL1*	rs1298634519	Y389 N	201	1038/0.85 (Probably damaging)	0.876/8/Disease	0.755/5/Disease	0.428/1/Neutral	0.956

For *SLCO1A2*, several transcript-specific substitutions, including G68R, G369R, D440Y, and D572Y, showed unanimous agreement on being pathogenic. Both PhD-SNP and SNP&GO predicted these mutations as “disease-related” with probabilities between 0.86 and 0.96, while PANTHER-PSEP consistently indicated a damaging effect with evolutionary preservation scores above 900 million years. The variants D440Y and G237R showed the highest predicted functional impact, represented by MutPred2 scores of 0.911 and 0.934, respectively, which may indicate that these substitutions compromise the efficiency of transmembrane transport and substrate recognition (Table 3).

Two deleterious variants of *HIF1AN*, D201H and D94H, uniformly produced damaging predictions in all models, particularly in PhD-SNP (0.87–0.88 probability) and MutPred2 (0.833–0.948 probability). These instances probably implicate impaired oxygen-sensing or hydroxylase-based regulatory mechanisms that could modulate HIF1α signaling in conditions of hypoxic stress (Table 3).

In *KCNQ3*, both incidences of the E170G variant possessed high pathogenic scores, as supported by PANTHER (0.927–0.941), PhD-SNP (0.942), and MutPred2 (0.912), and thus might alter ion channel conductance or gating. Lastly, the *DLL1* variant Y389 N presented with moderately damaging tendencies, supported by the highly conservative nature predicted by PANTHER-PSEP at 1038 million years and a MutPred2 score of 0.956, although SNP&GO suggested a neutral outcome (Table 3). Collectively, the convergence of predictions across multiple *in silico* frameworks supports the pathogenic relevance of these rare coding variants. Consistently high probability and reliability indices point toward the potential of these mutations to disrupt critical protein functions involved in the progression of EC and provide a justification for further structural and experimental validation studies.

### 3.7 Sequence-based prediction of the effects of rare-coding, deleterious non-synonymous SNPs on protein stability and function in EC biomarker genes

The sequence-based computational algorithms employed to investigate the effect of transcript-level rare-coding nsSNPs on the stability and function of the proteins were I-Mutant, MUpro, INPS, DDGun, and iStable. The ΔΔG values of the predicted stability changes and functional effects of these changes are listed in Table 4. Among the filtered EC biomarker genes, *GRB7*, *SLCO1A2*, *HIF1AN*, *KCNQ3*, and *DLL1* hosted variants with considerably altered protein stability. Among the prediction models, most variants had negative ΔΔG values that were in agreement with decreased protein stability (Table 4).

**Table 4:** Sequence-based prediction of the effects of rare-coding deleterious non-synonymous SNPs on protein stability and function in EC biomarker genes.

Gene	Variant ID	Position	Transcript ID	I Mutant (ΔΔG kcal/mol)	MUpro (ΔΔG)	INPS (ΔΔG)	DDGun		iStable (ΔΔG)
							Blosum score	(ΔΔG)	
*GRB7*	rs1439495576	W454C	205	−0.88	−0.95	−1.68466	−12.458	−4.8	−0.7
*GRB7*	rs772287804	G457W	205	−0.04	−0.68	−0.74051	−6.154	0.2	0.51
*GRB7*	rs764104340	R461H	205	−0.47	−0.96	−1.02569	−4.756	−0.8	−0.6
*GRB7*	rs764104340	R461L	205	0.17	0.08	1.07503	−6.782	−1.2	0.75
*GRB7*	rs778186811	R481Q	205	−0.99	0.009	−1.19216	−5.055	−1.1	−0.6
*SLCO1A2*	rs1943447924	D572Y	201	0.07	−0.23	−0.05468	−5.003	−0.1	0.768
*SLCO1A2*	rs1056174384	G369R	201	−0.94	−1.22	−0.2105	−5.682	−0.6	−0.738
*SLCO1A2*	rs1591837395	G68R	201	−2	−0.45	−0.25735	−3.189	0.1	0.635
*SLCO1A2*	rs1591837395	G68R	205	−2	−0.45	−0.54145	−5.385	−0.3	0.635
*SLCO1A2*	rs1591837395	G68R	207	−2	−0.45	−0.57289	−5.494	−0.3	0.635
*SLCO1A2*	rs1591837395	G66R	208	−1.64	−0.45	−0.55822	−5.596	−0.3	0.635
*SLCO1A2*	rs1591837395	G68R	209	−2	−0.45	−0.57289	−5.333	−0.2	0.635
*SLCO1A2*	rs1591837395	G68R	210	−2	−0.45	−0.52256	−5.337	−0.3	0.635
*SLCO1A2*	rs1943447924	D440Y	211	0.07	−0.24	−0.23671	−8.621	−0.5	0.768
*SLCO1A2*	rs1056174384	G237R	211	−1.22	−0.94	−0.37581	−7.746	−1.2	−0.738
*SLCO1A2*	rs1943447924	D572Y	217	0.07	−0.24	−0.05468	−5.003	−0.1	0.768
*SLCO1A2*	rs1056174384	G369R	217	−0.94	−1.21	−0.2105	−5.682	−0.6	−0.738
*SLCO1A2*	rs1591837395	G68R	217	−2	−0.45	−0.25735	−3.189	0.1	0.635
*HIF1AN*	rs758624092	D201H	201	0.24	−0.519051	−0.0942	−9.145	−0.9	−0.702
*HIF1AN*	rs758624092	D94H	205	0.24	−0.519051	−0.20348	−9.347	−0.9	−0.702
*KCNQ3*	rs777833596	E170G	201	−0.61	−1.456665	−0.8721	−5.646	−1.3	−0.575
*KCNQ3*	rs777833596	E170G	202	−0.61	−1.456665	−0.8721	−5.515	−1.3	−0.575
*DLL1*	rs1298634519	Y389 N	201	−1.69	−0.548912	−1.95942	−3.811	−2	−0.62865

The predicted and computed values of ΔΔG having negative sign indicate a decrease in the protein's stability.

Within *GRB7*, the variants W454C (rs1439495576), R481Q (rs778186811), and R461H (rs764104340) were reliably predicted by most algorithms as destabilizing substitutions with ΔΔG values between −0.47 and −12.45 kcal/mol. These substitutions fall within functionally important domains of *GRB7*, implying potential consequences on structural conformation and interface interactions in downstream signaling. In contrast, the R461L variant exhibited a mild tendency for stabilization as predicted by I-Mutant and MUpro but was predicted to be destabilizing by DDGun alone, which may reflect a context-dependent structural effect given the collective results of both datasets (Table 4).

For *SLCO1A2*, several transcript-specific mutations, most noticeably G68R (rs1591837395) and G369R (rs1056174384), returned substantially negative ΔΔG values across all prediction tools as low as −5.68 kcal/mol, pointing toward strong destabilizing effects likely to compromise transporter function. Interestingly, D572Y and D440Y had near-neutral ΔΔG values in I-Mutant and MUpro but were predicted to be destabilized by DDGun, suggesting subtle but possibly functionally relevant changes in local folding or interaction dynamics. The recurrence of G68R across multiple transcript isoforms underlines its potential pathogenic role in *SLCO1A2*-related alterations observed in EC (Table 4). Regarding *HIF1AN*, the D201H and D94H variants (rs758624092) were predicted to moderately reduce protein stability, with DDGun values of −9.3 kcal/mol. Such destabilization may interfere with the hydroxylase activity of HIF1AN and affect the hypoxia-associated pathways implicated in tumor progression (Table 4).

For *KCNQ3*, the E170G (rs777833596) variant was predicted to be deleterious by all five algorithms used. ΔΔG values were consistently negative, especially for MUpro (−1.45 kcal/mol) and DDGun (−5.64 kcal/mol). This suggests a disruption in the structural integrity of the voltage-gated potassium channel, which can impede ion transport (Table 4).

Finally, the *DLL1* variant Y389 N (rs1298634519) showed the most significant destabilization, with ΔΔG values as low as −1.96 for INPS and −3.81 for DDGun (Table 4). This mutation may affect ligand–receptor interactions in the Notch signaling pathway and alter cellular differentiation signals relevant to esophageal carcinogenesis. Overall, the concordant predictions of multiple algorithms suggest a uniform interpretation: most rare deleterious nsSNPs identified at the transcript level are associated with reduced protein stability. These destabilizing substitutions may disrupt protein folding, binding affinity, or enzymatic activity, contributing to the molecular dysregulation that is typical of EC.

### 3.8 Analysis for evolutionary conservation and HOPE server-based inference of EC-related rare-coding, deleterious nsSNPs

To assess the evolutionary significance of the identified rare-coding deleterious nonsynonymous variants, a conservation analysis was conducted using the ConSurf server with Bayesian-based conservation scoring. The results can be summarized by referring to Table 5. The conservation scores are observed in the range of 7-9, indicating that the most affected residues are highly conserved and likely to play important structural or functional roles within the corresponding proteins.

**Table 5:** Evolutionary conservation analysis and HOPE server-based structural impact assessment of rare-coding deleterious non-synonymous SNPs in EC-related genes.

Gene	Variant ID	Transcript ID	Conservation Profile	Residue Change Characteristics	Structural and Functional Implications
			Consurf Score/aBNC score (CI) (Conservation Inference)		
*GRB7*	rs1439495576 (W454C)	205	−1.049/9 (−1.183 to −1.002) (Highly conserved and buried)	Mutation introduces a smaller residue at a buried site	Mutation likely creates internal cavity within SH2 domain, weakening domain stability and signaling capacity
*GRB7*	rs772287804 (G457W)	205	−0.573/7 (−0.769 to −0.426) (Moderately conserved, buried)	Replacement of flexible glycine with bulky aromatic residue which is more hydrophobic than the wild-type residue	The mutation is expected to restrict backbone flexibility and distort local SH2 fold
*GRB7*	rs764104340 (R461H)	205	−1.141/9 (−1.191 to −1.118) (Highly conserved and surface exposed)	Loss of positive charge and reduced side-chain length	The mutation may disrupt intermolecular contacts essential for SH2-mediated interactions
*GRB7*	rs764104340 (R461L)	205	−1.141/9 (−1.191 to −1.118) (Highly conserved and surface exposed)	Charge removal and increased hydrophobicity	Predicted reduction in electrostatic interactions and altered protein binding
*GRB7*	rs778186811 (R481Q)	205	−1.141/9 (−1.191 to −1.118) (Highly conserved and exposed)	Neutralization of charged residue involved in H-bonding	Likely destabilizes local hydrogen bond network within SH2 region
*SLCO1A2*	rs1943447924 (D572Y)	201	−1.141/9 (−1.191 to −1.259) (Highly conserved and exposed)	Charge loss and increased hydrophobic bulk	Potential interference with surface interactions and protein folding
*SLCO1A2*	rs1056174384 (G369R)	201	−1.201/9 (−1.302 to −1.138) (Highly conserved and membrane buried)	Introduction of charged residue in TM region	Mutation may impair membrane embedding and transporter stability
*SLCO1A2*	rs1591837395 (G68R)	201	−1.201/9 (−1.302 to −1.138) (Highly conserved and TM buried)	Glycine replaced by bulky, charged arginine	Predicted disruption of transmembrane packing and transport efficiency
*SLCO1A2*	rs1591837395 (G68R)	205	−0.998/8 (−1.132 to −0.916) (Highly conserved and TM buried)	Glycine replaced by bulky, charged arginine	Predicted disruption of transmembrane packing and transport efficiency
*SLCO1A2*	rs1591837395 (G68R)	207	−1.038/9 (−1.180 to −0.955) (Highly conserved and TM buried)	Glycine replaced by bulky, charged arginine	Predicted disruption of transmembrane packing and transport efficiency
*SLCO1A2*	rs1591837395 (G66R)	208	−1.097/9 (−1.161 to −1.075) (Highly conserved and buried)	Increased steric hindrance in MFS (Major facilitator superfamily) domain	Likely compromises transporter conformational flexibility
*SLCO1A2*	rs1591837395 (G68R)	209	−0.889/8 (−1.021 to −0.793) (Highly conserved and TM buried)	Glycine replaced by bulky, charged arginine	Predicted disruption of transmembrane packing and transport efficiency
*SLCO1A2*	rs1591837395 (G68R)	210	−0.927/8 (−1.062 to −0.873) (Highly conserved and TM buried)	Glycine replaced by bulky, charged arginine	Predicted disruption of transmembrane packing and transport efficiency
*SLCO1A2*	rs1943447924 (D440Y)	211	−1.245/9 (−1.286 to −1.239) (Highly conserved and exposed)	Replacement of a negatively charged residue with a larger, neutral and more hydrophobic amino acid	Loss of electrostatic interactions and increased steric bulk may disrupt hydrogen bonding and impair surface stability or folding
*SLCO1A2*	rs1056174384 (G237R)	211	−1.166/9 (−1.270 to −1.139) (Highly conserved and buried)	Substitution of flexible glycine with a bulky, positively charged residue	Introduction of charge and reduced flexibility at a buried site may destabilize the protein core and affect transporter function
*SLCO1A2*	rs1943447924 (D572Y)	217	−1.277/9 (−1.328 to −1.259) (Highly conserved and exposed)	Loss of acidic residue replaced by larger hydrophobic amino acid	Altered surface charge and increased hydrophobicity may weaken intermolecular interactions and local structural integrity
*SLCO1A2*	rs1056174384 (G369R)	217	−1.201/9 (−1.302 to −1.138) (Highly conserved, TM buried)	Introduction of a bulky, positively charged residue within a transmembrane region	Likely disruption of lipid interactions and membrane packing, reducing transport efficiency
*SLCO1A2*	rs1591837395 (G68R)	217	−1.191/9 (−1.302 to −1.138) (Highly conserved, TM buried)	Replacement of small, flexible glycine with charged arginine	Reduced helix flexibility and disturbed membrane embedding may compromise protein stability and substrate translocation
*HIF1AN*	rs758624092 (D201H)	201	−0.885/9 (−0.939 to −0.866) (Highly conserved and exposed)	Charge neutralization at conserved site	May weaken regulatory interactions involved in hypoxia signaling
*HIF1AN*	rs758624092 (D94H)	205	−1.232/9 (−1.309 to −1.202) (Highly conserved and exposed)	Replacement alters electrostatic environment	Expected to affect protein stability and enzymatic regulation
*KCNQ3*	rs777833596 (E170G)	201	−0.959/9 (−0.991 to −0.946) (Highly conserved and TM buried)	Loss of charged residue and increased flexibility	Likely destabilizes ion channel structure and gating behavior
*KCNQ3*	rs777833596 (E170G)	202	−0.957/9 (−0.989 to −0.949) (Highly conserved and TM buried)		
*DLL1*	rs1298634519 (Y389 N)	201	−0.721/7 (−0.912 to −0.641) (Moderately conserved and exposed)	Reduced hydrophobicity and size	Potential loss of surface interactions involved in Notch signaling

a BNC=Bayesian method for calculating rates with a CI (Confidence Interval) to each of the inferred evolutionary conservation scores. The amino acids with scores between 7 and 9 were evolutionary conservative amino acids.

Among these, W454C (rs1439495576), R461H (rs764104340), R461L (rs764104340), and R481Q (rs778186811) are highly conserved residues located in the protein core or at functionally critical sites. Substitutions here would likely risk structural perturbations or disruption of binding interactions critical for *GRB7*-mediated signaling. The G457W variant, although moderately conserved, appears to be structurally sensitive because a flexible glycine has been substituted with a bulky aromatic residue (Table 5).

The HOPE-based structural interpretation supported these findings. The W454C substitution replaces a large aromatic residue with a much smaller side chain, creating internal voids that can destabilize the SH2 domain of the protein. The G457W mutation abolishes the backbone flexibility provided by glycine, probably by imposing inappropriate torsion angles and disrupting local folding. The R461H and R461L variants entail the loss of a positive charge at an exposed residue, likely disrupting intermolecular contacts and weakening *GRB7* interactions with signaling partners. The R481Q substitution abolishes the positive charge and diminishes the side-chain size, probably impairing hydrogen bond stabilization and compromising SH2 domain integrity (Table 5).

Most variants in *SLCO1A2*, such as G68R (rs1591837395), G369R (rs1056174384), and D440Y (rs1943447924), displayed high conservation scores (8-9), suggesting a strong evolutionary constraint. Several of these residues are buried, suggesting a maintenance role in the transmembrane architecture. The highly conserved but exposed D572Y variant may perturb substrate association and membrane contacts. The recurrence of G68R across multiple transcript isoforms implies its possible biological relevance (Table 5).

HOPE analysis showed that various *SLCO1A2* changes introduce a larger positive residue for a small or neutral residue, which may influence membrane packing, hydrophobic interactions, and conformational flexibility, which are important for the stability of a transporter. The loss of a negative charge in variants such as D440Y and D572Y could interfere with hydrogen-bond networks and proper folding, increasing the likelihood of impaired function (Table 5) The D94H and D201H substitutions are predicted to occur at highly conserved, exposed residues for *HIF1AN*. HOPE inferred that replacing a negatively charged aspartate with a larger neutral histidine may disrupt local packing and eliminate stabilizing charge interactions, potentially altering the oxygen-sensing mechanisms regulated by *HIF1AN* (Table 5).

The *KCNQ3* variant E170G, located in a buried region of the transmembrane domain, showed the highest conservation score (9). HOPE indicated that replacing glutamate with flexible glycine may compromise the structural rigidity needed for ion channel gating and disturb interactions with membrane lipids (Table 5).

In *DLL1*, the Y389 N variant showed moderate conservation (score 7) and was exposed on the surface. HOPE predicted that the loss of residue bulk and hydrophobicity may weaken the structural contacts important for Notch ligand-receptor binding (Table 5).

In particular, the integration of evolutionary conservation and HOPE-based structural inference demonstrated that most deleterious nsSNPs affect residues of high functional significance. Many substitutions are predicted to lead to steric clashes, loss of charge, altered hydrophobicity, or reduced flexibility, which may collectively disturb folding, stability, molecular interactions, and domain structure (Table 5). These insights support the potential pathogenic role of these variants in the molecular processes underlying esophageal cancer.

### 3.9 Analysis of surface accessibility, secondary structure, disorder, and torsion angles of wild-type and mutant proteins

The structural consequences of the identified rare-coding deleterious nsSNPs were further explored through computational analysis of surface accessibility, secondary structure elements, disorder probability, and dihedral torsion angles (Φ and Ψ). The comparative results for both the wild-type and mutant residues are summarized in Table 6.

**Table 6:** Computation of surface accessibility, secondary structure, disorderness and dihedral torsion angles of wild type and rare-coding non-synonymous mutations of EC genomic markers.

Gene	Variant ID	AA	Transcript ID	*Wild Type*							*Mutant Type*						
				RSA (%)	ASA (A^0^)	SS_3_	SS_8_	Phi	Psi	P_disorder (%)_	RSA (%)	ASA (A^0^)	SS_3_	SS_8_	Phi	Psi	P_disorder (%)_
*GRB7*	rs1439495576	W454C	205	8	19	Coil	Turn	−90	52	0	7	10	Coil	Coil	−91	55	1
*GRB7*	rs772287804	G457W	205	21	17	Coil	Bend	96	−147	0	25	60	Coil	Turn	17	−27	1
*GRB7*	rs764104340	R461H	205	29	67	Helix	Alpha-helix	−58	−42	0	30	55	Helix	Alpha-helix	−59	−42	0
*GRB7*	rs764104340	R461L	205								24	44	Helix	Alpha-helix	−58	−42	0
*GRB7*	rs778186811	R481Q	205	10	23	Strand	Beta-sheet	−126	140	0	10	19	Strand	Beta-sheet	−126	140	0
*SLCO1A2*	rs1943447924	D572Y	201	7	10	Helix	Alpha-helix	−66	−41	0	9	20	Helix	Alpha-helix	−64	−42	0
*SLCO1A2*	rs1056174384	G369R	201	0	0	Helix	Alpha-helix	−65	−40	0	1	3	Helix	Alpha-helix	−66	−39	0
*SLCO1A2*	rs1591837395	G68R	201	5	4	Helix	Alpha-helix	−65	−40	0	14	32	Helix	Alpha-helix	−67	−37	0
*SLCO1A2*	rs1591837395	G68R	205	7	5	Helix	Alpha-helix	−64	−43	0	28	64	Helix	Alpha-helix	−66	−39	0
*SLCO1A2*	rs1591837395	G68R	207	25	19	Helix	Alpha-helix	−64	−42	0	48	111	Helix	Alpha-helix	−66	40	0
*SLCO1A2*	rs1591837395	G66R	208	25	20	Helix	Alpha-helix	−64	−38	1	49	112	Helix	Alpha-helix	−65	−37	1
*SLCO1A2*	rs1591837395	G68R	209	25	19	Helix	Alpha-helix	−64	−43	0	50	113	Helix	Alpha-helix	−66	−40	0
*SLCO1A2*	rs1591837395	G68R	210	7	5	Helix	Alpha-helix	−64	−43	0	25	57	Helix	Alpha-helix	−66	−40	0
*SLCO1A2*	rs1943447924	D440Y	211	6	9	Helix	Alpha-helix	−64	−41	0	14	30	Helix	Alpha-helix	−67	−40	0
*SLCO1A2*	rs1056174384	G237R	211	0	0	Helix	Alpha-helix	−64	−41	0	1	3	Helix	Alpha-helix	−66	−38	0
*SLCO1A2*	rs1943447924	D572Y	217	7	10	Helix	Alpha-helix	−66	−41	0	9	20	Helix	Alpha-helix	−64	−42	0
*SLCO1A2*	rs1056174384	G369R	217	0	0	Helix	Alpha-helix	−65	−40	0	1	3	Helix	Alpha-helix	−66	−39	0
*SLCO1A2*	rs1591837395	G68R	217	5	4	Helix	Alpha-helix	−65	−40	0	14	32	Helix	Alpha-helix	−67	−37	0
*HIF1AN*	rs758624092	D201H	201	7	10	Coil	Coil	−107	139	0	8	15	Coil	Coil	−109	134	0
*HIF1AN*	rs758624092	D94H	205	12	18	Coil	Coil	−110	140	0	14	26	Coil	Coil	−111	134	0
*KCNQ3*	rs777833596	E170G	201	3	5	Helix	Alpha-helix	−66	−40	0	2	2	Helix	Alpha-helix	−65	−40	0
*KCNQ3*	rs777833596	E170G	202	3	5	Helix	Alpha-helix	−66	−40	0	2	1	Helix	Alpha-helix	−65	−40	0
*DLL1*	rs1298634519	Y389 N	201	17	35	Strand	Beta-sheet	−109	139	1	27	40	Coil	Beta-sheet	−93	98	2

For the *GRB7* variants, mutations such as W454C, G457W, R461 H/L, and R481Q exhibited notable differences in the relative solvent accessibility (RSA) and accessible surface area (ASA) between the wild-type and mutant forms. The W454C mutation, for example, showed a slight reduction in solvent exposure and a shift in the secondary structure from a turn to a coil, which could affect local flexibility. The G457W variant demonstrated a marked increase in surface accessibility (from 21% to 25%), reflecting a possible alteration in side-chain exposure that might influence molecular interactions. However, the R461H and R481Q substitutions retained their alpha-helical and beta-strand conformations, respectively, suggesting that although the backbone geometry remained stable, side-chain changes could still perturb the protein microenvironment (Table 6).

In the *SLCO1A2* gene, several transcript-specific variants (notably G68R, G369R, and D572Y) exhibited consistent structural alterations. The G68R variant across multiple transcripts displayed a considerable increase in RSA and ASA values, indicating enhanced solvent exposure and potential local unfolding within the α-helical region. These changes were accompanied by minor variations in the torsion angles, suggesting a subtle distortion of the helical geometry. The D440Y and D572Y substitutions also showed increased ASA and surface exposure in the mutant forms, which could affect the transmembrane stability or substrate transport efficiency. The overall low predicted disorder values (Pdisorder ≤1%) suggest that these mutations likely occur in structured regions rather than in intrinsically disordered domains (Table 6).

For *HIF1AN*, both D94H and D201H variants showed minor increases in surface exposure (RSA: 7–8%) and maintained their helical conformations. The similarity in torsion angles (Φ and Ψ) between the wild-type and mutant forms implies limited backbone rearrangement, although slight variations may still influence the protein-protein interaction surfaces involved in oxygen-sensing mechanisms (Table 6).

The *KCNQ3* variant E170G, observed in two transcripts, remained within the helical region, with minimal structural deviation. The changes in RSA and ASA in Table 6 are minimal, indicating that the destabilizing impact of this mutation is likely due primarily to the change in side-chain charge rather than significant structural rearrangements.

In *DLL1*, the Y389 N variant also showed a remarkable conformational change, from a beta-sheet-associated strand in the wild type to a coil region in the mutant. This was accompanied by increased solvent exposure (RSA increasing from 17% to 27%) and a higher probability of disorder, suggesting local unfolding or disruption of structural order. Such a modification could potentially impair Notch receptor binding, a critical function of *DLL1* within the pathways of cellular differentiation (Table 6).

The comparative analysis performed in this work indicates that most rare-coding deleterious nsSNPs induce local perturbations in surface exposure and secondary structure, mostly in regions that are important for the structural stability or molecular recognition of the protein. Although the global fold is frequently preserved, subtle changes in accessibility, backbone torsion angle variations, and secondary structure composition point toward plausible mechanisms by which these variants may influence protein stability and function in the pathogenesis of esophageal cancer.

### 3.10 Predicted effects of amino acid substitutions on proteins’ stability function of rare-coding, deleterious nsSNPs in EC biomarkers variants

To examine the effects that the identified missense variants could have on protein behavior, we analyzed the stability changes predicted by DynaMut along with complementary analyses provided through ENCoM, mCSM, SDM, and DUET. Among the EC-associated proteins, most of the substitutions presented negative ΔΔG values, thus reflecting a loss of stability and an overall destabilizing mutation. Vibrational entropy values were useful in elucidating whether each mutation could cause an increase or a decrease in molecular flexibility (Table 7).

**Table 7:** Predicted effects of rare-coding, deleterious nsSNPs on proteins’ stability function using DynaMut and normal mode analysis in EC biomarkers variants.

Genes	Mutations	Position	Transcript ID	DynaMut ΔΔG (kcal/mol)	NMA ENCoM ΔΔG (kcal/mol)	mCSM ΔΔG (kcal/mol)	SDM ΔΔG (kcal/mol)	DUET ΔΔG (kcal/mol)	ΔΔS Vib ENCoM (kcal.mol^−1^.K^−1^)
*GRB7*	rs1439495576	W454C	205	−1.023	−0.979	−1.68	−1.55	−1.596	1.224^a^
*GRB7*	rs772287804	G457W	205	0.971	0.714	−1.394	−2.22	−1.758	−0.893^b^
*GRB7*	rs764104340	R461H	205	−0.05	0.144	−1.28	0.12	−1.098	−0.18^b^
*GRB7*	rs764104340	R461L	205	0.329	−0.141	0.25	0.7	0.609	0.176^a^
*GRB7*	rs778186811	R481Q	205	−0.345	−0.195	−1.497	−1.56	−1.684	0.244^a^
*SLCO1A2*	rs1943447924	D572Y	201	−0.51	−0.163	−0.757	−1.17	−0.996	0.203^a^
*SLCO1A2*	rs1056174384	G369R	201	0.45	1.594	−0.917	−1.94	−0.878	−1.992^b^
*SLCO1A2*	rs1591837395	G68R	201	−0.659	1.367	−1.411	−2.450	−1.435	−1.709^b^
*SLCO1A2*	rs1591837395	G68R	205	1.681	1.525	−0.49	0.86	−0.068	−1.907^b^
*SLCO1A2*	rs1591837395	G68R	207	0.029	−0.048	−0.534	0.86	−0.108	0.06^a^
*SLCO1A2*	rs1591837395	G66R	208	0.098	−0.028	−0.568	0.86	−0.139	0.035^a^
*SLCO1A2*	rs1591837395	G68R	209	0.073	−0.02	−0.535	0.86	−0.109	0.025^a^
*SLCO1A2*	rs1591837395	G68R	210	1.829	1.21	−0.51	1.04	−0.064	−1.512^b^
*SLCO1A2*	rs1943447924	D440Y	211	2.206	0.542	−0.153	0.26	0.016	−0.678^b^
*SLCO1A2*	rs1056174384	G237R	211	−0.017	0.609	−0.917	−2.230	−0.92	−0.762^b^
*SLCO1A2*	rs1943447924	D572Y	217	−0.51	−0.163	−0.757	−1.17	−0.996	0.203^a^
*SLCO1A2*	rs1056174384	G369R	217	0.45	1.594	−0.917	−1.94	−0.878	−1.992^b^
*SLCO1A2*	rs1591837395	G68R	217	−0.659	1.367	−1.411	−2.45	−1.435	−1.709^b^
*HIF1AN*	rs758624092	D201H	201	0.422	0.157	−1.563	0.83	−1.15	−0.196^b^
*HIF1AN*	rs758624092	D94H	205	0.458	0.116	−1.496	0.83	−1.089	−0.144^b^
*KCNQ3*	rs777833596	E170G	201	−0.523	−0.656	−0.995	−1.54	−1.173	0.82^a^
*KCNQ3*	rs777833596	E170G	202	−0.523	−0.656	−0.995	−1.540	−1.173	0.82^a^
*DLL1*	rs1298634519	Y389 N	201	−1.249	−0.951	−1.73	−2.33	−1.927	1.188^a^

a Indicates an increase in molecular flexibility.

b Indicates a decrease in molecular flexibility. NMA refers to normal mode analysis. ENCoM is the elastic network contact model. mCSM is the mutation cut-off scanning matrix. SDM is the site-directed mutator. ΔΔS Vib ENCoM represents the predicted change in vibrational entropy between the wild-type and mutant structures. Negative ΔΔG values reflect a loss of stability, while positive values indicate stabilizing effects.

A number of *GRB7* variants showed clear destabilizing trends. Substitutions W454C, R481Q, and R461H generally resulted in negative ΔΔG values in the majority of the prediction models, indicating reduced stability. W454C is consistently destabilizing across all methods; R461H yielded mixed results but tended toward being unstable. In contrast, R461L gave positive ΔΔG values in a number of tools, which may indicate a stabilizing effect. Flexibility predictions varied, with W454C and R481Q associated with increased flexibility, whereas G457W and R461H were predicted to reduce flexibility (Table 7).

*SLCO1A2* contains several mutations that recur in the different transcripts studied, most of which tend to destabilize the protein. Variant D572Y consistently exhibited negative ΔΔG values across all transcripts, indicating decreased stability coupled with increased molecular flexibility. Substitutions G369R and G68R were consistently destabilizing across mCSM, SDM, and DUET, regardless of the positive values returned by DynaMut or ENCoM. Flexibility predictions for these sites frequently indicate decreased flexibility, perhaps reflecting local structural tightening in the context of overall destabilization. Few predictions specific to transcripts, such as G68R at positions 205 and 210, produced positive ΔΔG values, suggesting context-dependent effects (Table 7).

The D201H and D94H variants of *HIF1AN* were predicted to decrease protein stability. The ΔΔG values from mCSM, SDM, and DUET were strongly negative for both mutations, and the vibrational entropy values indicated reduced molecular flexibility. Collectively, these results indicate a consistent destabilizing effect of these variants on the *HIF1AN* structure (Table 7).

The E170G substitution in *KCNQ3*, assessed at two transcript positions, had a consistent destabilizing pattern for all methods predicting ΔΔG. However, despite the decrease in stability, both entries resulted in increased flexibility, indicating loosening within the local structural regions (Table 7).

The Y389 N variant of *DLL1* had a significant destabilizing effect. All ΔΔG values from the tools were negative, indicating that the mutation increased molecular flexibility and resulted in a notable weakening of the *DLL1* protein structure (Table 7). Taken together, the majority of substitutions in *GRB7*, *SLCO1A2*, *HIF1AN*, *KCNQ3*, and *DLL1* demonstrated destabilizing patterns, with negative ΔΔG values and increased flexibility. Minor substitutions, like *GRB7* R461L and some transcript-dependent substitutions in *SLCO1A2*, were observed to have stabilizing tendencies as well (Table 7). These predictions shed light into the structural sensitivities of these selected esophageal cancer markers and indicate that several of their rare coding variants may disrupt protein stability in manners that could affect functional behavior.

### 3.11 Structural flexibility variations in mutant and wild-type protein models of EC-related rare-coding deleterious nsSNPs

The protein flexibility of all the chosen transcript-level variants was investigated using CABS-Flex 3 server to determine how every deleterious substitution alters mobility within the structure. Comparing RMSF values between wild-type and mutant structures, it can be observed that several variants produce substantial changes in flexibility whereas others affect more localized sites without overall structural changes.

**Fig. 6:**
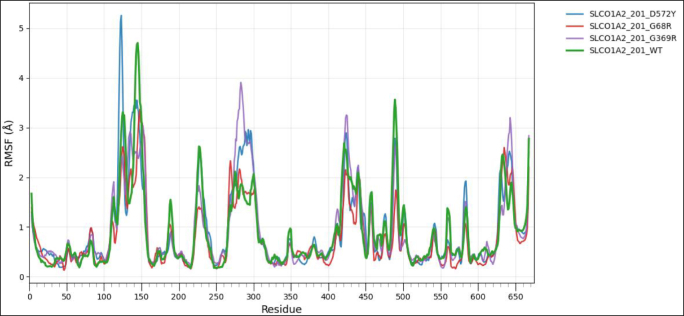
Root mean square fluctuation (RMSF) analysis comparing the wild-type SCLOA12 protein with mutant models of its rare-coding deleterious variants (transcript 201).

**Fig. 7:**
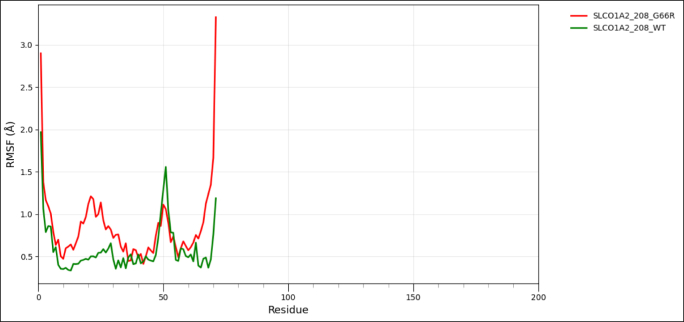
Root mean square fluctuation (RMSF) analysis comparing the wild-type SCLOA12 protein with mutant models of its rare-coding deleterious variants (transcript 208).

**Fig. 8:**
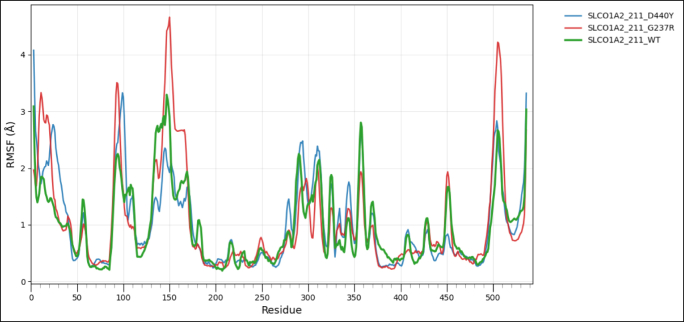
Root mean square fluctuation (RMSF) analysis comparing the wild-type SCLOA12 protein with mutant models of its rare-coding deleterious variants (transcript 211).

**Fig. 9:**
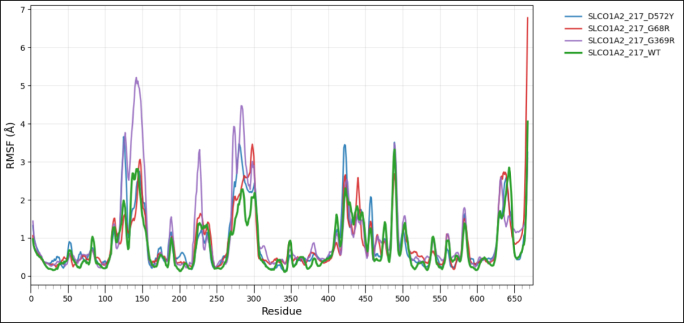
Root mean square fluctuation (RMSF) analysis comparing the wild-type SCLOA12 protein with mutant models of its rare-coding deleterious variants (transcript 217).

Among these *SLCO1A2* variants, the G68R substitution was the most strongly varying across transcripts. In transcript 201, G68R induced a strong increase in flexibility (RMSF from 0.239 to 0.767), which likely correlates with displacement or loosening of the protein environment in that region (Fig. 6). By contrast, in transcripts 205, 207, 209, and 210, the same amino acid substitution led to a decrease in mobility, indicating that the impact of this mutation is strongly dependent on the context of the specific transcript. The G66R variant also showed a clear gain in flexibility (from 0.473 to 0.904), whereas D440Y and D572Y generally reduced overall mobility, indicative of a stabilizing effect (Fig. 7). Mutations at positions G369R and G237R resulted in minor changes, hardly affecting the backbone dynamics (Fig. 8, Fig. 9).

Most of the variants showed a reduction in flexibility for *GRB7* compared to the wild type, such as W454C, R461H, R461L, and R481Q variants. A significant drop in RMSF from 1.019 to 0.277 for R481Q suggests that the local structure is tightened and may impact the *GRB7* interaction sites. G457W showed only a slight reduction, and this bulkier substitution may retain its dynamic profile in this region.

**Fig. 10:**
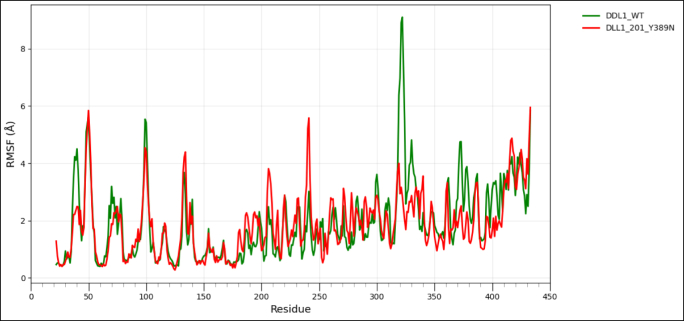
Root mean square fluctuation (RMSF) analysis comparing the wild-type DLL1 protein with mutant models of its rare-coding deleterious variants (transcript 201).

**Fig. 11:**
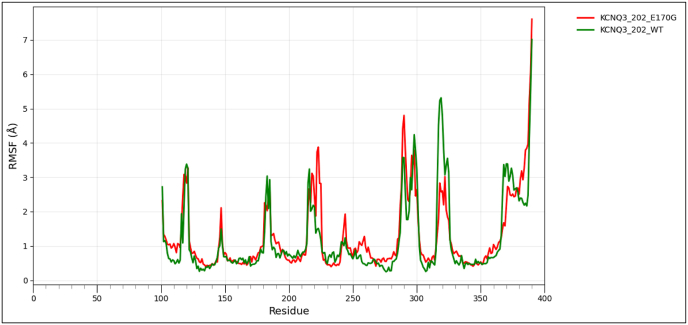
Root mean square fluctuation (RMSF) analysis comparing the wild-type KCNQ3 protein with mutant models of its rare-coding deleterious variants (transcript 202).

The *DLL1* variant Y389 N resulted in a slight increase in RMSF, from 1.365 to 1.467, indicating a modest destabilizing effect at this position in the helical region (Fig. 10). The two *HIF1AN* variants, D201H and D94H, both showed significant reductions in flexibility, suggesting that these substitutions could limit the natural motions required for optimal hydroxylase function and substrate interactions. In *KCNQ3*, the E170G mutation acted differentially in a transcript-dependent manner. In transcript 201, it reduced RMSF from 0.908 to 0.476, while in transcript 202, it enhanced the flexibility from 0.417 to 0.688. This contrast suggests that the same single point mutation may flip the channel dynamics in both directions depending on the structural context of the isoform (Fig. 11).

In all, flexibility analysis showed that the rare-coding pathogenic variants do not follow a single underlying pattern. Some mutations lead to increased motion and potential destabilization, while others restrict movement and may interfere with the conformational changes required for proper function. These dynamic patterns and signatures support earlier sequence and structure-based predictions and highlight the diverse ways in which rare-coding nsSNPs can alter protein behavior in EC-related genes.

### 3.12 Integrated structural and oncogenic predictions for rare-coding deleterious EC mutations

To understand how rare-coding missense variants may influence protein behavior and their likelihood of contributing to malignancy, we evaluated each substitution using several stability-prediction tools (mCSM, SDM, DUET, CUPSAT, and DynaMut2) and assessed their oncogenic potential using CScape. Across all genes, most variants produced negative ΔΔG values, indicating a loss of stability. The CScape scores also showed that nearly all substitutions fell within the oncogenic range, including several high-confidence calls (Table 8).

**Table 8:**
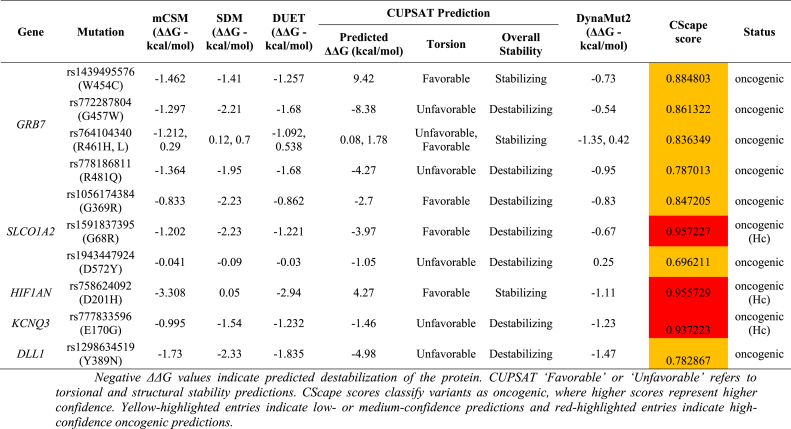
Predicted effects of rare-coding deleterious nsSNPs on protein stability and oncogenic potential in EC biomarkers genes.

All variants were predicted as oncogenic for *GRB7*. For the substitutions W454C, G457W, and R481Q, the ΔΔG values were consistently negative across methods, indicating destabilization of the protein. The CScape scores for these variants were in the range of 0.78 to 0.88, placing them squarely in the category of an oncogenic variant. The combined entry for R461H/R461L showed mixed effects on stability, with the substitution of R461H being destabilizing, while for the leucine replacement (R461L), stabilizing values were obtained in several models. However, the overall CScape score still supported its oncogenic status (Table 8).

The *SLCO1A2* variants consistently showed a tendency to be less stable. The G369R and G68R gave ΔΔG values that were considerably negative in most modeling systems, such as DUET and SDM, suggesting these substitutions would significantly destabilize the structure. Both of these variants were classified as oncogenic, while G68R was categorized as having high-confidence oncogenic properties. In contrast, the mutation D572Y had a somewhat weaker pattern of destabilizing effects, with some models predicting neutral or slightly stabilizing effects; however, the CScape score remained within the range for oncogenic changes (Table 8).

The D201H variant of *HIF1AN* exhibited a mixed stability profile, with a stabilizing prediction from SDM but strong destabilization predictions from mCSM and DUET. Despite these divergent assessments, a CScape score of 0.95 classified this mutation as a high-confidence oncogenic variant (Table 8). This finding implies that the mutation may impair *HIF1AN* function, even when local stability appears to be partially maintained.

In *KCNQ3*, the E170G substitution produced a uniform destabilization across all stability prediction tools. Negative ΔΔG estimates from mCSM, SDM, DUET, and DynaMut2 indicate structural weakening of the channel protein. The CScape score indicated a high-confidence oncogenic potential (Table 8).

The Y389 N variant in *DLL1* demonstrated strongly negative ΔΔG predictions across all models and was labeled oncogenic by CScape. The combined destabilizing and oncogenic patterns suggest that this substitution could impact Notch signaling in esophageal tissue (Table 8).

The combined stability and oncogenic predictions showed that nearly all rare-coding missense variants assessed across *GRB7*, *SLCO1A2*, *HIF1AN*, *KCNQ3*, and *DLL1* were both structurally disruptive and predicted to be oncogenic (Table 8). Variants with the strongest destabilizing signatures tended to have high CScape scores, supporting the idea that structural weakening may contribute to functional impairment relevant to esophageal cancer biology.

## 4 Discussion

This study employed a comprehensive computational framework to identify and characterize rare-coding pathogenic nsSNPs in genes associated with EC. This multilayered analysis integrated population genetics, sequence-based predictions, evolutionary conservation, and structural modeling to filter a set of high-confidence variants with the potential to disrupt the protein function and contribute to oncogenesis. This systematic approach, from a broad genomic landscape to specific structural consequences, provides a robust framework for elucidating the influence of rare coding variations on EC pathology [28,67].

Our initial analysis of 28 EC-related genes revealed a heterogeneous landscape of mutations. The large range in total and exonic single-nucleotide polymorphism counts with notably high variant densities in genes such as *KCNQ3* and *SLCO1A2* underlines the genomic complexity typical of EC [68,69]. This most likely is indicative of the differences in gene size, functional constraint, and different selective pressures. That genes responsible for transport and structural stability are among the ones with the highest number of exonic variants, *SLCO1A2* and *DMBT1*, might indicate a generally higher tolerance of pathways against more mutations or that their dysregulation presents a common event in EC, leading to an accumulation of a large number of passenger mutations [70,71]. The list of genes with a relatively lower number of variants, including *RNF187* and *CRNN*, points toward putative regions under strong purifying selection, where changes are less tolerated and, when occurring, may have more drastic functional implications [72,73].

The central result identifies ten rare-coding nonsynonymous single-nucleotide polymorphisms corresponding to 23 transcript-specific variants distributed across five filtered genes (*GRB7, SLCO1A2, HIF1AN, KCNQ3,* and *DLL1*), which were invariable predicted to be deleterious by a consensus of functional prediction tools such as SIFT, PolyPhen-2, CADD, and REVEL. Rarity is remarkable for all these variants (MAF <1%), thus fitting into a model in which only severe, highly penetrant risk alleles for complex diseases (such as cancer) can be maintained at low population frequencies [34]. The clustering of several disruptive variants within specific genes, particularly *GRB7* and *SLCO1A2*, highlights these genes as functional disruption hotspots [70,74]. Transcript-level analysis proved to be indispensable; it was shown that one SNP may occur in several isoforms, for instance, *SLCO1A2* G68R, which enhances its biological effect and indicates that any therapeutic or diagnostic strategy against this variant must consider this isoform heterogeneity [[75], [76], [77], [78]].

These (tissue-expression) expression patterns of the genes provide an essential biological context for the genetic findings reported herein. Markedly increased expression of *GRB7* and *DLL1* in the esophageal mucosa, the primary site of origin for both major EC subtypes, strongly endorses their functional relevance in this tissue [74,79,80]. *GRB7* is an extensively characterized adaptor protein implicated in growth factor signaling and cell migration [[81], [82], [83]], and its overexpression has been associated with tumor aggressiveness across multiple cancers [84]. Hence, the concentrated mucosal expression of GRB7 makes it a plausible driver of epithelial-specific oncogenic events in esophageal cancer [74,83,84]. Similarly, pronounced mucosal expression of *DLL1*, the ligand of the Notch signaling pathway, is noteworthy given the pivotal role of Notch in cell fate determination and differentiation as well as its context-dependent function as an oncogene or tumor suppressor [80]. On the other hand, the broad and moderate expression of *HIF1AN* across all esophageal tissues mirrors its fundamental role in oxygen sensing-a process central to tumor metabolism and progression [85,86].

Consensus classification of our variant prioritization employed PredictSNP, followed by sequence-based analyses using PANTHER-PSEP, PhD-SNP, and MutPred2. High conservation scores, often exceeding 900 million years in the case of affected residues, provide strong evolutionary evidence for positions of functional importance [43]. Given this, one would strongly assert that a residue conserved across such large evolutionary timescales is likely to be intolerant to substitution and, by corollary, alterations at these positions within our dataset strongly suggest a pathogenic outcome [43]. Also, MutPred2 suggested mechanistic disruptions such as gain or loss of ordered structures, alteration of interaction interfaces, or changes at enzymatic active sites, thus affording testable hypotheses for subsequent experimental validation [46].

One of the core findings of our stability analysis is that the majority of the identified rare-coding nsSNPs are destabilizing. The consistently negative ΔΔG values predicted by tools like I-Mutant, MUpro, and DDGun were an indication that such amino acid substitutions compromise the structural integrity of the corresponding proteins. For instance, the recurrent *SLCO1A2* G68R and G369R variants were repeatedly predicted to reduce protein stability. The substitution of a small, flexible glycine with the large, positively charged arginine within a transmembrane domain, such as in G68R, probably causes steric clashes and impairs the tight packing necessary for proper membrane protein function [87,88]. Such destabilization may result in protein misfolding and accelerate degradation, leading to the loss of the transporter function of *SLCO1A2*. This may impair the uptake of endogenous compounds or drugs, altering the tumor microenvironment or the therapeutic response [87,88].

HOPE and ConSurf analyses provided the residue-level interpretation for the observed destabilizing effects. The inferences all gave the indication of adverse biophysical changes, including loss of charge in *GRB7* R461 H/L and *SLCO1A2* D572Y, which could disrupt salt bridges or hydrogen bonds; introduction of bulky residues leading to steric hindrance; and substitutions that modify local hydrophobicity, with potential impacts on core packing or membrane integration [[89], [90], [91], [92]]. That many deleterious variants are localized to important functional domains directly associates structural perturbation with an expected loss of molecular function, whether it be at the level of signal transduction, ion transport, or substrate recognition, such as the SH2 domain of *GRB7* or the transmembrane helices of *SLCO1A2* and *KCNQ3* [[89], [90], [91], [92]].

Importantly, given our results based on DynaMut and CABS-Flex on structural dynamics, our results have implications that extend beyond static stability alone [59,60]. These predictions of changes in vibrational entropy and residue fluctuation (RMSF) suggest that such mutations may affect the dynamic properties of the protein [59,60,89,[91], [92], [93]]. Variants such as *GRB7* W454C increase flexibility and, therefore, could destabilize a structured domain, impairing specific binding [60,92,94]. Others reduce flexibility, possibly by rigidifying the protein and impeding the conformational changes necessary for activity [60,91,94]. For *KCNQ3*, a potassium channel, such dynamic changes can directly influence the gating mechanics and ion conductance [68,89,91,92,95]. The observed transcript-dependent differences in flexibility for the same mutation shown by *SLCO1A2* G68R emphasized that the structural context of an isoform can fundamentally alter the functional outcome of a genetic variant [30,96,97].

Integration with stability predictions and oncogenic potential via CScape provides a robust linkage between molecular structural perturbation and oncogenesis [66]. That almost all identified shortlisted variants were classified as oncogenic, and three of these, *SLCO1A2* G68R, *HIF1AN* D201H, and *KCNQ3* E170G, were accorded high-confidence assessments, strongly suggests that these variants serve as possible drivers of esophageal tumorigenesis rather than incidental polymorphisms [68,78,86,95]. Among the latter, particular notice may be taken of the *HIF1AN* D201H variant. *HIF1AN* is the key regulator of the master transcription factor HIF1α, through which the cell orchestrates its response to hypoxia [85,86]. A destabilizing mutation such as D201H that impairs the function of *HIF1AN* could result in constitutive HIF1α activity under normoxic conditions, thus facilitating angiogenesis, metabolic reprogramming, and metastatic processes in aggressive cancer [85,86,98,99]. A second clear path to oncogenesis involves disruption of DLL1, a ligand within the Notch signaling pathway, which may disrupt cell differentiation and proliferation programmes in the esophagus epithelium [80,100].

## 5 Conclusion

This work presented a set of rare-coding deleterious nsSNPs within five filtered EC-related genes, using a systematic computational workflow that integrated variant filtering, functional prediction, evolutionary conservation analysis, and structural evaluation. The present study demonstrated how these variants could compromise the stability, folding, and molecular interaction networks of proteins at the core of pathways associated with the progression of EC. Such strong concordance among multiple predictive tools, combined with supporting evidence of conservation and HOPE-based structural interpretation, suggests the potential biological relevance of these variants. Although experimental validation remains necessary, these findings provide a focused set of candidate variants that may contribute to the understanding of the molecular mechanisms underlying esophageal carcinogenesis and may help guide future biomarker or therapeutic investigations.

## Funding

The author(s) declare that no funding support was received for the research.

## CRediT authorship contribution statement

**Muhammad Bilal Azmi:** Conceptualization, Data curation, Formal analysis, Investigation, Methodology, Project administration, Resources, Software, Supervision, Validation, Writing – original draft, Writing – review & editing. **Sajida Qureshi:** Conceptualization, Formal analysis, Funding acquisition, Investigation, Methodology, Project administration, Resources, Validation, Writing – original draft, Writing – review & editing. **Rafia Wasi:** Data curation, Formal analysis, Investigation, Methodology, Software, Validation, Visualization, Writing – original draft, Writing – review & editing. **Saad Khalid Niaz:** Formal analysis, Funding acquisition, Methodology, Resources, Writing – original draft, Writing – review & editing. **Syed Danish Haseen Ahmed:** Formal analysis, Funding acquisition, Project administration, Resources, Validation, Writing – original draft, Writing – review & editing.

## Declaration of competing interest

The authors declare that they have no known competing financial interests or personal relationships that could have appeared to influence the work reported in this paper.

## Data Availability

All data generated or analyzed during this study are included in this manuscript [and its supplementary material files].
