# Supplementary material for: Computational identification of rare pathogenic genomic variants in esophageal cancer markers: Transcript-level analysis, sequence-based insights, and structural-functional impacts of non-synonymous SNPs
Source: Biochem Biophys Rep. 2026 Feb 23;45:102503. doi: 10.1016/j.bbrep.2026.102503 (PMC12952785; doi:10.1016/j.bbrep.2026.102503)
Supplement: Multimedia component 1 [file mmc1.zip › S3 Table.docx]

**S3 Table. Table showing gene-tissues expression details of filtered genes biomarkers of Esophageal Cancers**

| **Genes** | **Esophageal Tissues (Median TPM / Sample)** | | |  |
| --- | --- | --- | --- | --- |
|  | **Esophagus - Gastroesophageal Junction (n = 403)** | **Esophagus - Mucosa (n = 614)** | **Esophagus - Muscularis (n= 561)** |  |
| *GRB7* | 0.2946 | 62.25 | 0.3144 |  |
|  |  |  |  |  |
|  |  |  |  |  |
|  |  |  |  |  |
| *SLCO1A2* | 0.00998 | 0.004625 | 0.00837 |  |
|  |  |  |  |  |
|  |  |  |  |  |
| *HIF1AN* | 13.35 | 9.503 | 13.65 |  |
| *KCNQ3* | 0.4155 | 0.1955 | 0.3585 |  |
| *DLL1* | 11.88 | 28.51 | 10.09 |  |
